# *Pho1a* (plastid starch phosphorylase) is duplicated and essential for normal starch granule phenotype in tubers of *Solanum tuberosum* L

**DOI:** 10.3389/fpls.2023.1220973

**Published:** 2023-08-09

**Authors:** Shrikant Sharma, Martin Friberg, Paul Vogel, Helle Turesson, Niklas Olsson, Mariette Andersson, Per Hofvander

**Affiliations:** Department of Plant Breeding, Swedish University of Agricultural Sciences, Alnarp, Sweden

**Keywords:** starch phosphorylase, reserve starch, sink tissue, starch granule, tuber, potato, CRISPR, gene editing

## Abstract

Reserve starch from seeds and tubers is a crucial plant product for human survival. Much research has been devoted to quantitative and qualitative aspects of starch synthesis and its relation to abiotic factors of importance in agriculture. Certain aspects of genetic factors and enzymes influencing carbon assimilation into starch granules remain elusive after many decades of research. Starch phosphorylase (Pho) can operate, depending on metabolic conditions, in a synthetic and degradative pathway. The plastidial form of the enzyme is one of the most highly expressed genes in potato tubers, and the encoded product is imported into starch-synthesizing amyloplasts. We identified that the genomic locus of a Pho1a-type starch phosphorylase is duplicated in potato. Our study further shows that the enzyme is of importance for a normal starch granule phenotype in tubers. Null mutants created by genome editing display rounded starch granules in an increased number that contained a reduced ratio of apparent amylose in the starch.

## 1 Introduction

Plant α-glucan phosphorylase or starch phosphorylase is a somewhat enigmatic enzyme where both cytosolic and plastid forms can be found that are encoded by independent genes (Mori et al., 1991; Sonnewald et al., 1995; Albrecht et al., 2001). The role of the enzyme forms has been investigated in several plant species over a long time with different findings regarding a role in biosynthetic or degradative pathways and importance of spatio/temporal or abiotic conditions that have been comprehensively reviewed (Rathore et al., 2009; Hwang et al., 2020; Li et al., 2021; Shoaib et al., 2021). The plastid directed *Pho1a* is one of the highest expressed genes in potato tuber (Sonnewald et al., 1995; Albrecht et al., 2001; Van Harsselaar et al., 2017), but its precise role regarding reserve starch accumulation, if any, remains elusive.

Starch phosphorylase (EC 2.4.1.1) is a member of the GT35-gycosyl transferase superfamily and carries out a transfer of glucosyl unit to or from an α-1,4-glucan chain in a reversible reaction (Hanes, 1940; Brisson et al., 1989; Rathore et al., 2009; Cuesta-Seijo et al., 2017). The release or incorporation of glucose-1-phosphate (G-1-P) is suggested to be dependent on multiple factors including the ratio of G-1-P to inorganic phosphate (Pi) (Preiss and Levi, 1980; Kruger and ap Rees, 1983; Schupp and Ziegler, 2004; Satoh et al., 2008; Rathore et al., 2009; Tiessen et al., 2012; Tetlow and Bertoft, 2020). Two forms of starch phosphorylase are reported in higher plants, localized to plastid (Pho1: L type) and cytosol, respectively (Pho2: H type) (Brisson et al., 1989; Sonnewald et al., 1995; Albrecht et al., 2001). The cellular compartmentalization of respective isozymes subjects them to different metabolic effectors, redox environments, and protein turnover factors (Albrecht et al., 1998; Albrecht et al., 2001; Hwang et al., 2020). In addition to a peptide for plastid localization, a major difference in Pho1 from Pho2 is an internal, approximately 78–82 amino acid domain (L80). This domain forms an extended auxiliary loop of unordered structure and is suggested to define substrate specificity based on stereological hindrance in binding to large polysaccharides (Nakano and Fukui, 1986; Albrecht et al., 1998; Chen et al., 2002; Young et al., 2006; Tickle et al., 2009; Hwang et al., 2016b; Cuesta-Seijo et al., 2017; Nakamura et al., 2017). The L80 domain was found not to be of any importance for catalytic functions of Pho1 in rice (Hwang et al., 2010). However, it contains a highly variable set of negatively charged amino acids, phosphorylation sites, and a PEST motif, which is reported to be a substrate of proteasomes to modulate the degradation of Pho1 in sweet potato (Chen et al., 2002; Young et al., 2006; Lin et al., 2012). The exact role of Pho isozymes in starch metabolism in higher plants has been debated over decades. Although it is generally accepted that the plastidial form of Pho1 is involved in maltooligosaccharides (MOs) metabolism, cytosolic Pho2 is generally involved in maltose metabolism resulting from starch degradation (Lu et al., 2006; Satoh et al., 2008; Hwang et al., 2010; Flores-Castellanos and Fettke, 2022).

Various studies have indicated that Pho1 is involved in transitory starch turnover in photosynthetic and reserve starch accumulation in sink organs of multiple species (cereal grains, roots, and tubers) by maintenance of plastidial maltodextrin pools (Chen et al., 2002; Schupp and Ziegler, 2004; Young et al., 2006; Subasinghe, 2013; Hwang et al., 2016b; Nakamura et al., 2017). In *Arabidopsis*, *PHS1*, a homolog of *Pho1* in *Arabidopsis*, is suggested to be part of the core set of evolutionary conserved genes, involved in starch granule initiation (Mérida and Fettke, 2021). Pho1 is also reported to form complexes with multiple starch synthases (SS) and starch-branching enzymes (SBEs) in several cereals including wheat, rice, maize, and barley, and has been shown to influence the starch synthesis (Tetlow et al., 2008; Liu et al., 2009; Nakamura et al., 2012; Ahmed et al., 2015; Crofts et al., 2017; Nakamura et al., 2017). Other complexes that have been reported are with disproportionating enzyme 1 (Dpe1, EC 2.4.1.25) in sweet potato and rice (Hwang et al., 2016a; Nakamura et al., 2017). The enzyme is suggested to serve in recycling of MOs released from the trimming of pre-amylopectin by debranching enzymes (DBEs) leading to the accumulation of G-1-P (Satoh et al., 2008; Hwang et al., 2010; Hwang et al., 2016a; Lin et al., 2017). The direct incorporation of G-1-P to the surface of native starch granules and soluble MOs in maltodextrin pool by Pho1 has been demonstrated in *in vitro* assays and with potato tuber disks (Fettke et al., 2010; Fettke et al., 2012; Flores-Castellanos and Fettke, 2022). Reserve starch accumulation in potato tubers has been suggested to follow two interacting pathways depending on the environmental conditions (Fettke et al., 2012). The high catalytic activity of Pho1 at lower temperatures as compared to ADP-glucose pyrophosphorylase (AGPase) has then been suggested to provide a complimentary pathway for starch biosynthesis at low temperatures in potato (Fettke et al., 2012; Slugina et al., 2020a; Mérida and Fettke, 2021).

Two isoforms of Pho1 are reported in potato, Pho1a and Pho1b (Brisson et al., 1989; Nakano et al., 1989; Sonnewald et al., 1995; Albrecht et al., 2001). The genes encoding the isoforms are suggested to be located on chromosome 3 and 5, respectively (Schreiber et al., 2014; Schönhals et al., 2016; Van Harsselaar et al., 2017; Slugina et al., 2020b) and localized on respective chromosomes in latest genome assemblies (Pham et al., 2020; Jayakody et al., 2023; Yang et al., 2023). The corresponding mRNAs are assembled from 15 and 14 exons, respectively, and an insertion of a 5,060-bp-long TE/Copia-like retrotransposon (*Tst1*) has been reported in the fifth intron of *Pho1a* (CamIrand et al., 1990). Both proteins are highly similar (81%–84%) in amino acid sequence identity, while the N-terminal transit peptide and L80 insertion domain are more diverse (Albrecht et al., 2001). Pho1a is reported to be ubiquitously present in both leaves and tubers, whereas the gene encoding Pho1b was mostly expressed in leaves and close vicinity to vascular tissue in tubers (Albrecht et al., 2001). Although expression of *Pho1b* is found to be higher in leaves and clearly detectable in tubers, at protein level, Pho1a is reported to be more abundant in leaves and tubers, whereas Pho1b was undetectable in tubers (Sonnewald et al., 1995; Albrecht et al., 1998; Albrecht et al., 2001). Pho1a has been detected as homo- (Pho1a)_2_ and hetero-dimer (Pho1a–Pho1b) in leaves, but only as homodimer in tubers (Albrecht et al., 1998; Albrecht et al., 2001).

In this study, we show that the *Pho1a* gene is tandemly duplicated in the potato genome. We show that the transposon *Tst1* likely is not the driver of this duplication, as species in *Solanaceae* with duplication and no *Tst1* insertion exists. Furthermore, we show that knocking out the *Pho1a* genes, using genome editing, affects the starch granule phenotype in reserve starch of potato tubers. A decrease in measured amylose to amylopectin ratio was observed that could indicate a changed chain length distribution affecting the observed granule phenotypical change. This shows that the major plastid starch phosphorylase activity of potato has a role in the organization of reserve starch structure but not for general starch accumulation capacity. No major differences in starch degradation into sugars could be found between null mutants and the parental variety upon cold storage of tubers.

## 2 Materials and methods

### 2.1 Plastid starch phosphorylase (*Pho1a*) mutagenesis

**Figure 1:**
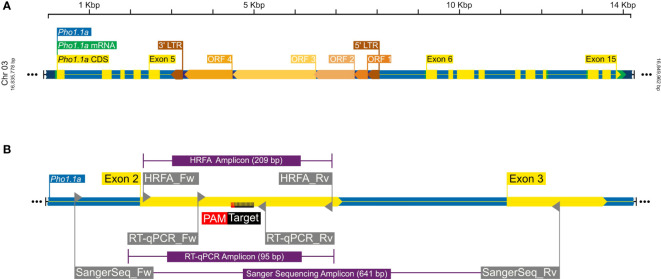
A graphical representation of *Pho1.1a* genomic loci. **(A)** Full-length *Pho1.1a* loci from *S. tuberosum* Group Phureja DM 1-3 516 R44 Genome Assembly (DM v6.1). **(B)** Enlarged view of *Pho1.1a* sequence representing location of Exon 2/3, RNP target adjacent to PAM sequence on negative strand, primers and respective amplicons for HFRA, Sanger sequencing, and RT-qPCR analysis. The sequences are color coded as follows: genomic as blue, mRNA as green, CDS/Exon as yellow, 5′ and 3′ LTRs as brown, ORFs of *Tst1* as shades of dark yellow, primers as gray, amplicons as purple, and RNP target/PAM site as black/red. The top scale represents length of *Pho1.1a* loci, and left/right numbers represent location of chromosome 3 of DM v6.1.

To determine a CRISPR target sequence, a partial genomic sequence of the plastidial starch phosphorylase was extracted from *Solanum tuberosum* cv. Desirée using primers listed in **Table 1** and with a method previously described (Zhao et al., 2021). The target sequence to induce mutations in the *Pho1a* gene (**Figure 1A**) was selected using the CRISPR RGEN Tools (http://www.rgenome.net/cas-designer). The selected target, 5′-GCTGTTGCAAAGAATGCCTT-3′, was located on the negative strand on exon 2, adjacent the PAM site 5′-AGG-3′ (**Figure 1B**). Leaf tissue from *in vitro* grown plants of *S. tuberosum* L. cv Desirée was used for protoplast isolation, transfection, and shoot regeneration as described previously (Nicolia et al., 2021). Purified protoplasts were transfected with preassembled ribonucleoprotein complexes (RNPs hereafter) of 5 µg of Cas9 enzyme (Thermo Fisher Scientific, Waltham, MA, USA) and 0.1 nmol sgRNA, (Synthego, Redwood city, CA, USA), using 40% PEG 4000 (Sigma-Aldrich, Germany) with an incubation time of 30 min. Regenerated shoot isolation was limited to one from each callus for further analysis.

**Table 1:** List of primers used in this study for HFRA analysis, Sanger sequencing, and RT-qPCR based copy number estimation.

Application	Primer code	Amplicon	Primer sequence (5′–3′)	T_A_ (°C)	Amplicon Size (bp)
HFRA Analysis	HFRA_Fw	Pho1a_E2	AGAGCGACCTGAGTTCTTTT-FAM	62	209
	HFRA_Rw		GTACGCTTGCTTCATGTTCA		
Sanger Sequencing and ICE analysis	SangerSeq_Fw	Pho1a_E2-E3	CAGAAACTTGATGTATGGATCTTAGG	62	641
	SangerSeq_Rv		GCACCAGTAAGCTCCAGATT		
RT-qPCR based copy number estimation and *GBSS1* expression	qStgbssF	*StGBSS_qRT*	TTGCATAACTGGGATTGTGAATG	52	93
	qStgbssR		GACAGTGGTTATATCGTATTTGACATCTG		
	RT-qPCR_Fw	*StPho1a_qRT*	CCATGCAGAATTCACACCTG	52	95
	RT-qPCR_Rv		TAAGGAGCGAATCACGAACA		
	qSttubF	*StTUBB1_qRT*	GTTGGCAATTCAACCTCCAT	60	143
	qSttubR		ATGTTGCTCTCGGCTTCAGT		
Targetted Cloning and sequencing	PHO1.1a_F1	Amplicon 1	GTTTTAATTTGCGAGAGAGAGAGAG	62	601
	PHO1.1a_R1		TCTGTGAATGCCATGTCAGC		
	PHO1.1a_F2	Amplicon 2	TAGGGAGATGGTCACTGTTCCAG	62	904
	PHO1.1a_R2		TCACATCCCTTCACTTGTTCCTG		
	PHO1.1a_F2	Amplicon 3	TAGGGAGATGGTCACTGTTCCAG	62	786
	PHO1.2a_R2		GTGCTAAGACAAGAAGGAAGGTG		
	PHO1.2a_F1	Amplicon 4	ACCACATAATAAGAGATGAAGAGTCTC	62	895
	PHO1.2a_R1		TCAAATAGCCTCGCACTTACTC		
	PHO1.2a_F2	Amplicon 5	GAAGCTCATCCAAGATGACTATCTG	62	807
	PHO1.2a_R2		GTGCTAAGACAAGAAGGAAGGTG		

**Figure 2:**
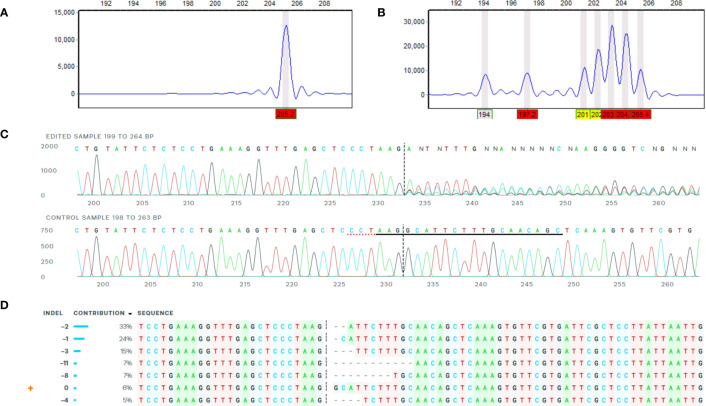
Example of confirmation of mutations by HFRA and ICE analysis. The figure represents HFRA and ICE analysis for confirmation of mutations in one representative mutational line, SPD-15 (1WT). **(A)** One peak detected in HFRA chromatogram representing the wild-type alleles in Desirée (control). **(B)** Seven peaks detected in HFRA chromatogram representing indels of seven different sizes in SPD-15. **(C)** Sanger sequencing trace data: top, SPD-15 and bottom, Desirée. The vertical dashed line represents Cas9 blunt cut, three nucleotides upstream from PAM site on negative strand. Red dashed line represents PAM site (AGG) on negative strand. Solid black line marks sgRNA guide (5′-GCTGTTGCAAAGAATGCCTT-3′) on negative strand. **(D)** Deduced sequence of six mutational and one wild-type alleles in SPD-15 and their relative abundance based on Sanger sequencing data. The calculated contribution indicate that two alleles have the same type of indel, either a −2 or a −1 deletion.

A primary analysis for screening of induced indels was made using high-resolution fragment analysis (HRFA hereafter) of PCR amplicons spanning the target site as described previously (Andersson et al., 2017) using primers listed in **Table 1** and marked in **Figure 1B**. The mutations were confirmed by Sanger sequencing of PCR amplicons using primers listed in **Table 1** and marked in **Figure 1B**, and indel distribution was analyzed using online ICE analysis (http://ice.synthego.com) (**Figure 2**). *In vitro* cuttings of selected events with indels (**Table 2**) and Desirée (as control) were planted in soil (Yrkesplantjord, SW Horto, Hammenhög, Sweden) as three to five biological replicates in 7.5-L pots. Plants were cultivated under controlled greenhouse conditions (16-h day length, 18/15°C day/night temperature, supplementary light intensity up to approximately 200 μmol s^−1^ m^−2^ photons, 50% relative humidity) for 5 months and were regularly fertilized with SW Bouyant RikaS 7-1-5 + mikro (SWHorto, Hammenhög, Sweden).

**Table 2:** Size of indels (bp) and mutational outcome in respective regenerated events.

Group	Line	Event ID	Size of indels (bp)	Mutational outcome
Group 1 (Full knockouts; FKO)	SPD-1	181057	-4;-2;-1	full knock, all alleles out of frame mutations
	SPD-2	181085	-5;-2;-1	
	SPD-3	181092	-2;-1	
	SPD-4	181113	-2;-1;1	
	SPD-5	181124	-10;-4;-2;-1	
Group 2 (In-Frame knockouts; IFM)	SPD-6	181004	-6;-5;-4;-2;-1	full knock, at least one allele in frame
	SPD-7	181011	-5;-3;-2;-1	
	SPD-8	181015	-6;-2;-1;	
	SPD-9	181027	-9;-5;-2;-1	
	SPD-10	181116	-8;-4;-3;-2;-1;1	
Group 3 (Partial knockouts; WTA)	SPD-11	181013	-16;-12;-2;0	events with at least one wild type allele
	SPD-12	181017	-9;-4;-2;-1;0	
	SPD-14	181065	-36;-12;-2;-1;0	
	SPD-15	181130	-11;-8;-4;-3;-2;-1;0	

“0” represents at least one WT allele present, and “−X” and “X” represents a deletion or insertion where X is the number of bp. The events have been sorted in three groups: full knockout and out of frame indels, all alleles with mutations but at least one allele has an in-frame indel, and events with mutations but at least one allele is wild type.

### 2.2 Characterization of *Pho1a* genomic loci

For the *in silico* characterization of the genomic *Pho1a* loci, a reference mRNA sequence of *Pho1a* (GenBank accession no. X52385.1) was blasted against high confidence gene models of long-read chromosome-scale genome assembly of doubled monoploid potato *S. tuberosum* Group Phureja DM 1-3 516 R44 Genome Assembly v6.1 (DM v6.1 hereafter) using blastn with default parameters (http://spuddb.uga.edu/blast.shtml) (Pham et al., 2020). Top hits above 99% identity and E-value 0.0 on chromosome 3 were identified as putative *Pho1a* loci in DM v6.1 assembly.

These putative *Pho1a* genomic sequences were aligned with previously reported *Pho1a* transcripts based on PGSC v4.03 assembly (Sharma et al., 2013; Van Harsselaar et al., 2017), *Pho1a* genomic sequence extracted from Desirée genomic DNA library (Zhao et al., 2021), and copia-like transposable element *Tst1* (CamIrand et al., 1990) using Clustal Omega (v1.2.2) at EMBL-EBI (https://www.ebi.ac.uk/Tools/msa/clustalo/) to refine exon/intron structure and deduce full length genomic sequence of *Pho1a* loci. Additionally, genomic sequences of homologous *Pho1a* loci from 24 wild potato species, representative of tuber-bearing clade of *Petota* section, two non-tuber-bearing wild potato species from the neighboring section *Etuberosum*, and two landraces of *S. tuberosum* Group Phureja (http://solomics.agis.org.cn/potato/tool/blast)(Priyam et al., 2019; Tang et al., 2022) and *S. lycopersicum* (http://spuddb.uga.edu/SollycM82_v1_download.shtml; (Alonge et al., 2022) were retrieved by blast search using reference mRNA sequence of *Pho1a*. These putative *Pho1a* genomic sequences from above assemblies were also aligned with above-mentioned complete *Pho1a* genomic sequence using Clustal Omega (v1.2.2) to detect the presence of duplication and insertion of *Tst1* in respective species. Specific genomic regions were amplified by PCR using primers listed in **Table 1** and subsequently cloned in pJET1.2 using Clone Jet PCR Cloning kit (Thermo Fisher Scientific, Waltham, MA, USA). The resulting clones with inserts were subjected to Sanger sequencing (LGC Genomics, Berlin, Germany) in triplicates to confirm organization of duplicated *Pho1a* loci on chromosome 3 of *S. tuberosum* L. cv Desirée.

The duplication of *Pho1a* in cv. Desirée based on HRFA and ICE analysis was examined by copy number estimation by Real Time quantitative PCR (RT-qPCR) on QuantStudio3 thermocycler (Applied Biosystems, USA) using Maxima SYBR Green/ROX qPCR Master Mix (2X) (K0221, Thermo Fisher Scientific, Waltham, MA, USA). *StGBSS1* (GenBank accession no. A23741.1), a known single-copy gene, was used as reference gene (Andersson et al., 2017). The primer pairs for *Pho1a* were selected to match *StGBSS1* primers in terms of amplicon length and T_m_ (**Table 1**) (Andersson et al., 2006). The optimal T_m_ was determined based on R^2^ closest to =1 from the standard curve of Desirée gDNA dilution series in duplicates (10, 1, 0.1, 0.01 and 0.001 ng) using VeriFlex (Applied Biosystems, USA), and the initial copy number in 10 ng gDNA sample was calculated using following formula:


$$X_{n}\;=X_{0}{\left(1+E\right)}_{n}$$

where *X_n_* is PCR product after cycle n, *X_0_* is initial copy number, *E* is amplification efficiency, and *n* is cycle number.

### 2.3 Phenotypic characterization

Plants were photographed at three time points during greenhouse cultivation (4 weeks, 12 weeks, and maturity) to compare growth rate characteristics and individual leaf samples from greenhouse cultivated events were collected (middle and end of light phase) to evaluate transitory starch characteristics. Briefly, the leaf samples were fixed by immersion into fixative solution (3.7% formaldehyde and 0.1 M phosphate buffer, pH 6.5) for 24 h at 37°C, dehydrated and decolored (50% (v/v) ethanol for 24 h, and 96% (v/v) ethanol for 2 × 24 h; both steps at 37°C) and stored at 4°C. Afterwards, the leaf samples were rehydrated (50% (v/v) ethanol for 30–60 min; dH_2_O for 20–30 min), stained with Lugol’s solution (2% KI (w/v) and 1% I_2_ (w/v) for 3 min), and visualized under a light microscope (Leica DMLB, Wetzlar, Germany) equipped with an Infinity X-32 digital camera (DeltaPix, Samourn, Denmark) as described previously (Ovecka et al., 2012).

The number, shape, and total fresh weight of the tubers were recorded at the time of harvest. A subset of freshly harvested tubers from each mutational line was sliced into halves. One was immediately flash frozen in liquid nitrogen and stored at −80°C for subsequent initial free sugar content analysis. The other was freeze-dried for 48 h and utilized to determine dry matter, total starch, and starch composition. The weight of freeze-dried tuber samples was recorded, and dry matter was calculated as described previously (Zhao et al., 2021). In addition, the freshly harvested tubers were also cross-sectioned along vertical and horizontal axis, stained with Lugol’s solution for 1 min, washed with dH_2_O, and photographed (Cannon 450D DSLR) on a light table to record distribution of starch granules across tuber axis(s) in triplicates. These tuber samples were further sliced into 0.5-mm-thin sections using a mandolin and visualized under a light microscope after staining with Lugol’s solution as previously described (Zhao et al., 2021). The size of starch granules were graded into three groups: a) small up to 25 µm, b) medium 25–50 µm, and c) large above 50 µm. The remaining tubers from each event were stored at 4°C for 3 months and used to determine free sugar levels in tubers after cold storage.

### 2.4 Starch phosphorylase activity staining

Tuber extracts from a subset of mutational events (based on above phenotypic characterization) and Desirée (control) were assessed for starch phosphorylase activity using a modified version of (Sweetloove et al., 1996). In total, nine mutational events and Desirée (as control) were selected for further analysis. Tuber samples from three biological replicates of each individual line were pooled and ground using a mortar and pestle in extraction buffer (100 mM HEPES, 10 mM EDTA, 5 mM DTT, 10% Glycerol, 0.1% PVPP, pH 7.5) and 1× Protease inhibitor cocktail for plant cell and tissue extracts (Sigma-Aldrich, USA), on ice. Extracts were centrifuged for 10 min at 14,000 *g*, at 4°C. Supernatant was loaded directly on a native PAGE gel (Novex, 10% Tris-Glycine, Invitrogen or 12% native PAGE cast with 0.8% glycogen). Novex gel was run for 120 min at 200 V at 4°C. The glycogen containing gel was run for 120 min at 250 V at 4°C. Subsequently, both gels were washed in an incubation buffer (100 mM Tris–HCl, 1 mM MgCl_2_, 1 mM CaCl_2_, 5% glycerol, pH 7) for 10 min before being moved to a substrate buffer (incubation buffer supplemented with 50 mM glucose-1-phosphate, 2.5 mM AMP) and incubated at room temperature (approximately 22°C) for 3 h. After incubation, the gel was briefly rinsed in dH_2_O before staining for 5–10 min in 0.1× Lugol’s solution and imaged using a photocopier (Epson Perfection V750 Pro, Seiko Epson Corporation, Suwa, Japan).

### 2.5 Determination of total starch content

Freeze-dried samples from five full knockout events were homogenized in a Retsch Mixer Mill MM400 (Retsch, Germany), at 30 Hz, for 30 s, and 50 mg of homogenized material was utilized to determine total starch content using Total Starch Assay Kit (K-TSTA-100A, Megazyme, Wicklow, Ireland) following the manufacturer’s instructions. Briefly, total starch in the samples was converted into maltodextrins by thermostable α-amylase (100°C, 15 min), which were subsequently quantitatively hydrolyzed into D-glucose by amyloglucosidase (50°C, 30 min). The resulting D-glucose was measured in a colorimetric reaction employing glucose oxidase/peroxidase (GOPOD) reagent, and absorbance was measured at 510 nm (Multiskan GO, Thermo Fisher Scientific, USA). All analysis were performed in triplicates; total starch concentration was calculated using Mega-Calc™ (Megazyme, Ireland) and reported as mean values of percentage on dry weight (DW) basis.

### 2.6 Determination of starch composition and *StGBSS1* expression

Amylose content was measured in starch from tubers of nine mutational events and Desirée (as control) by a colorimetric method, as described previously (Chrastil, 1987). In short, 150 mg of homogenized material from freeze-dried tuber samples was suspended in 70% EtOH. The suspension was sieved through a nylon mesh to remove debris, and total starch was pelleted by centrifugation (2,000 *g*, 20 min). The supernatant was discarded, and the pellet was dried overnight. For each sample, 8 mg of purified starch was used for analysis. Samples were suspended in water, solubilized with 5M NaOH, and incubated at room temperature with intense agitation for 1.5 h. After solubilization, samples were neutralized with 3M HCl and then buffered by addition of 50mM sodium phosphate buffer. Samples were stained using iodine dissolved in 85% Dimethyl sulfoxide (DMSO), incubated for 10 min at room temperature, and the absorbance was measured at 620 and 550 nm (Multiskan GO, Thermo Fisher Scientific, USA). Amylose content was calculated as percent of total starch content using the following formula.


$$190.2R^{2}-\;\left(281.52R\right)+106.6$$

where $R=A_{620nm}/A_{550nm}$ The expression level of *StGBSS1* was estimated in tuber samples of selected events by RT-qPCR on a QuantStudio3 thermocycler (Applied Biosystems, USA) using Maxima SYBR Green/ROX qPCR Master Mix (2X) (K0221, Thermo Fisher Scientific, Waltham, MA, USA), using primers listed in **Table 1**, and *StTUBB1* (NM_001288449.1) was used as reference gene as described above in *Section 2.2*. The samples were run in triplicates, and the fold change was calculated by 2^–δδCt^ as described previously (Livak and Schmittgen, 2001).

### 2.7 Determination of free sugars levels

The effect of Pho1a starch phosphorylase presence on starch degradation was determined by estimation of free sugars levels, i.e., sucrose, fructose, and glucose in flash-frozen and cold-stored tuber samples using Sucrose/D-Fructose/D-Glucose Assay Kit (K-SUFRG, Megazyme, Wicklow, Ireland) following the manufacturer’s instructions. A total of 50 mg of homogenized material from flash-frozen tubers samples was utilized to determine the initial free sugar content in tuber samples at the time of harvest. The free sugar analysis was repeated on 50 mg of homogenized tuber samples, from tubers stored at 4°C for 3 months. In brief, the assay involved pH-dependent conversion of D-glucose and D-fructose into glucose-6-phosphate (G-6-P) intermediates using a hexokinase/phosphoglucose isomerase/glucose-6-phosphate dehydrogenase-based reaction and subsequent stoichiometric quantification of Nicotinamide adenine dinucleotide phosphate (NADPH) by absorbance at 340 nm (light path: 1 cm; ~25°C). The sucrose level was calculated from the difference in D-glucose concentration before and after the hydrolysis by β-fructosidase. D-Glucose and sucrose standards were used to ensure accuracy of spectrophotometer measurements and effectiveness of the β-fructosidase hydrolysis reaction by comparing D-glucose to D-fructose ratio, respectively. All analyses were performed in triplicates; free sugar levels were calculated using Mega-Calc™ (Megazyme, Ireland) and reported as mean values of percentage on fresh weight (FW) basis.

## 3 Results

### 3.1 *Pho1a* mutagenesis yield more than four variant alleles in tetraploid potato

Mutations were induced in exon 2 of the *Pho1a* gene in the auto-tetraploid potato cv. Desirée. In total, 140 regenerated events were selected for HRFA screening for indels, and 129 out of the 140 events had induced mutations in at least one allele (92%) (data not shown). A total of 14 events, 5 events from Group 1 (full knockouts; FKO), 5 events from Group 2 (in-frame mutation; IFM), and 4 events from Group 3 (containing wild-type alleles; WTA), were selected for greenhouse cultivation and further characterization (**Table 2**, **Figure 2**, and **Supplementary Figure S3**). These selected mutational events, i.e., Starch Phosphorylase mutational events in Desirée, were termed as SPD-1 to SPD-15 in sequential order. A wide variation in allelic dosage of mutations was detected, and allelic variants in individual events spanned from one to seven. The presence of up to seven mutated alleles was confirmed by ICE analysis, and copy number of the gene was estimated to be 2.23 relative to 1 for a confirmed single-copy gene, *StGBSS1* by RT-qPCR-based copy number estimation. Taken together, these results indicated that the *Pho1a* locus is duplicated in cultivated tetraploid potato cv Desirée.

### 3.2 The *Pho1a* gene is duplicated in potato

**Figure 3:**
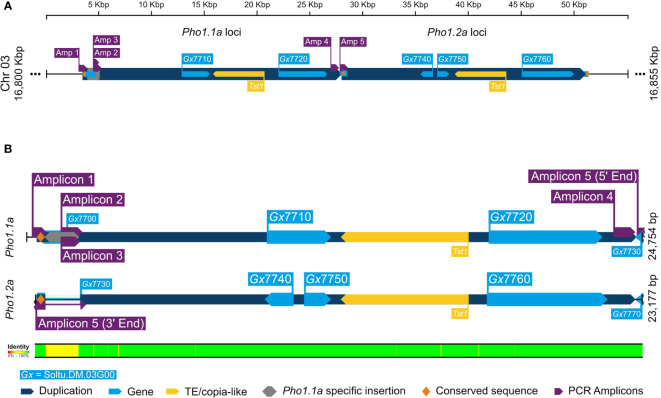
A graphical representation of alignment of duplicated *Pho1a* genomic loci in DM v6.1. **(A)** The duplicated genomic segments as dark blue blocks and gene annotations in DM v6.1 as light blue blocks. **(B)** Sequence alignment of *Pho1.1a* and *Pho1.2a* loci. The targeted cloning and sequencing amplicons are in purple, *Pho1.1a*-specific insertion sequence in gray, and conserved repeating sequence as yellow. The respective sequence IDs are annotated, and sequence identity conservation is represented as color coded legend at the bottom.

The duplication of *Pho1a* and its genomic organization in the current DM v6.1 genome assembly was further confirmed by *in silico* analysis and targeted sequencing. A blast search for reference mRNA sequence of *Pho1a* (X52385.1) against DM v6.1 genome assembly resulted in five hits on chromosome 3 with high sequence identity, namely, Soltu.DM.03G007710 to Soltu.DM.03G007760 (**Supplementary Table S1**). The cDNA sequence of Soltu.DM.03G007710 shared high sequence homology with the first part of *Pho1a* mRNA, whereas Soltu.DM.03G007720 was highly similar to the distal part of reference mRNA sequence of *Pho1a* (X52385.1) (**Supplementary Figure S1**). Similarly, DNA sequence of Soltu.DM.03G007740 (annotated on reverse strand) and Soltu.DM.03G007750 were highly similar to the first part of *Pho1a* mRNA, whereas Soltu.DM.03G007760 was similar to the distal part (**Supplementary Figure S1**). The first part would contain predicted exons 1–5, while the distal part would contain predicted exons 6–15. In addition, a previously reported copia-like transposable element *Tst1* (X52287.1) was found to align with the genomic sequence between Soltu.DM.03G007710 and Soltu.DM.03G007720, and Soltu.DM.03G007750 and Soltu.DM.03G007760 with high similarity (**Supplementary Figure S1**). Based on sequence homology, these two genomic segments were considered to be duplicated *Pho1a* loci and termed *Pho1.1a* (i.e., Soltu.DM.03G007710 and Soltu.DM.03G007720) and *Pho1.2a* (i.e., Soltu.DM.03G007740 to Soltu.DM.03G007760) loci. Furthermore, the genomic sequence up to 7.7 kbp upstream of *Pho1.1a* and *Pho1.2a* coding sequence was nearly identical (**Figure 3**). However, a further 1.7 kbp upstream genomic region consisted of a 1.3-kbp insertional segment specific to the *Pho1.1a* locus. These two highly similar genomic regions, i.e., approximately 25 kbp region (Chr03:16803346.16827878) covering *Pho1.1a* and approximately 23 kbp region (Chr03:16827879.16851055) encompassing *Pho1.2a*, were delimited by approximately 300-bp-long conserved sequence, which may have served as recombination hotspots (**Figure 3**). The genomic assembly of both *Pho1a* loci was confirmed by targeted PCR amplification, cloning, and Sanger sequencing (**Figure 3**). Cloned sequences from amplicons 1 to 3 mapped specifically to the *Pho1.1a* locus, whereas amplicon 5 sequence was specific for the *Pho1.2a* locus (**Figure 3**). Amplicon 4 sequence, on the other hand, mapped to the end of exon 15 of *Pho1.1a* locus into the upstream region of the *Pho1.2a* locus, thus confirming the sequential order of *Pho1.1a* and *Pho1.2a* loci in the DM v6.1 genome assembly (**Figure 3**).

### 3.3 The *Pho1a* duplication is independent of the *Tst1* insertion

**Figure 4:**
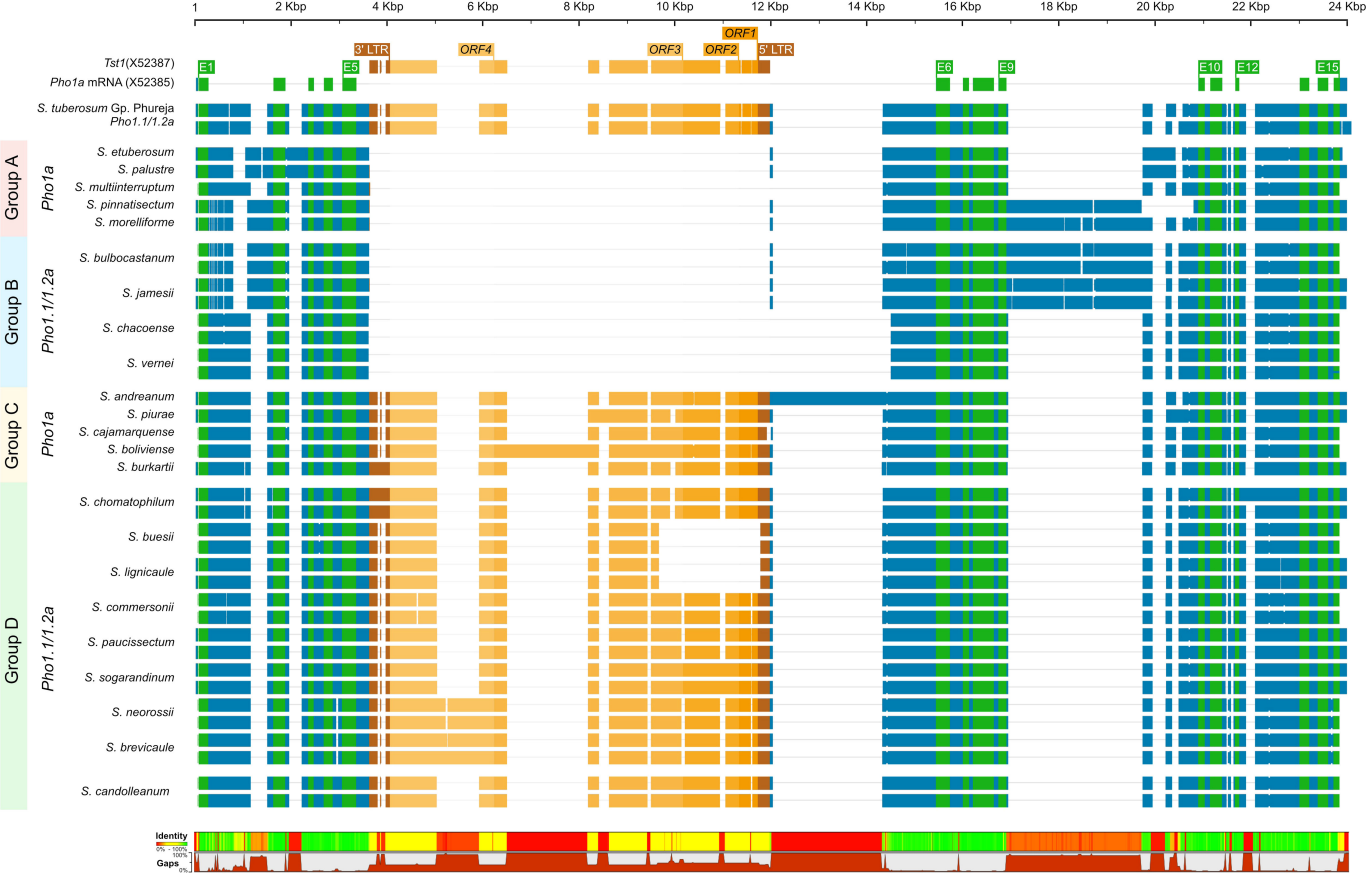
A graphical representation of alignment of *Pho1.1a* and *Pho1.2a* genomic sequences from diploid assemblies of wild and cultivated potato species from *Petota* and *Etuberosum* section. The species are divided into four groups based on presence of *Tst1* and duplication of *Pho1a* loci. Group 1: no duplication and insertion of *Tst1*; Group 2: duplicated *Pho1a* loci and insertion of *Tst1*; Group 3: No duplication and with insertion of *Tst1*; Group 2: duplicated *Pho1a* loci and with insertion of *Tst1.* The sequences are color coded as per: genomic as blue, mRNA as green, CDS/Exon as yellow, 5′ and 3′ LTRs as brown, and ORFs of *Tst1* as shades of dark yellow. The top scale represents length of single/duplicated *Pho1a* loci, and bottom bar chart represents sequence identity and gaps. The alignment was generated with Clustal Omega.

The origins of the *Tst1* insertion and its possible role in duplication were investigated by *in silico* analysis of putative *Pho1a* genomic loci in recently released genome assemblies of two non-tuber-bearing wild potato species from *Etuberosum* section, 24 diploid wild potato species from tuber-bearing clade of *Petota* section, 2 landraces of *S. tuberosum* Group Phureja and tomato cv. M82. A blast search, using reference mRNA sequence of *Pho1a* (X52385.1) against the above-mentioned genome assemblies resulted in the identification of putative 1 or 2 *Pho1a* loci in respective assemblies (**Supplementary Table S2**). When compared to 13,716 bp in DM v6.1, the length of *Pho1a* loci in the above assemblies was variable, ranging from 8,440 to 16,228 bp, depending on the presence of insertion and the length of *Tst1* in intron 5 and species-specific insertions in introns 2, 9, 11, and 13 (**Figure 4**; **Supplementary Table S2**). However, the length of duplicated *Pho1a* loci, i.e., *Pho1.1a* and *Pho1.2a* were nearly identical in individual species (**Figure 4**; **Supplementary Table S2**). The duplication of *Pho1a* was found to be independent of insertion of *Tst1*, as both single and duplicated *Pho1a* loci were detected with and without a *Tst1* insertion in potato species (**Figure 4**; **Supplementary Table S2**). On the other hand, a single *Pho1a* locus without *Tst1* insertion was detected in both potato species from *Etuberosum* section and tomato, which suggests that the insertion of *Tst1* in *Pho1a* may be specific to certain clades of *Petota* section in *Solanaceae* (**Figure 4**; **Supplementary Table S2**). As a result, the above potato species could be organized into four groups, namely, 1) single *Pho1a* locus without insertion of *Tst1*, 2) single *Pho1a* locus with insertion of *Tst1*, 3) duplicated *Pho1a* loci without insertion of *Tst1*, and 4) duplicated *Pho1a* loci with insertion of *Tst1* (**Figure 4**).

### 3.4 *Pho1a* knockout results in an increased number of tubers

**Figure 5:**
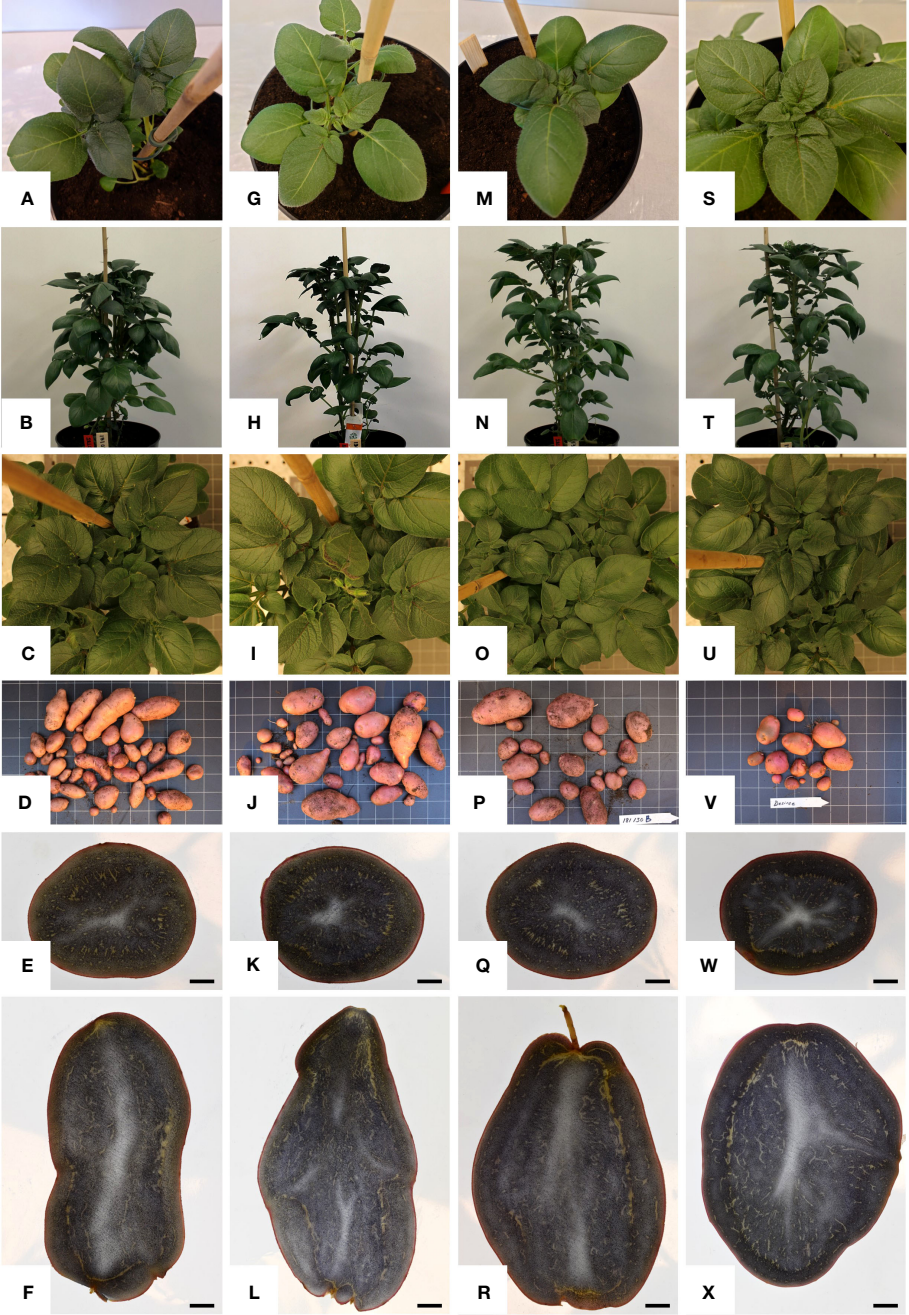
Phenotypic characterization of mutational events selected for greenhouse trial. One representative from each group, i.e., from left: column 1, **(A–F)** SPD-1: Group 1 (full knockouts; FKO); column 2, **(G–I)** SPD-6: Group 2 (in-frame knockouts; IFM); Column 3, **(M–R)** SPD-15 Group 3 (partial knockouts; WTA), and column 4, **(S–X)** Desirée (WT, control). First row: **(A, G, M, S)** represent top view of 2 weeks old plants; second row: **(B, H, N, T)** represent front view of 4-week-old plants; and third row: **(C, I, O, U)** represent top view of 4-week-old plants. Row 4: **(D, J, P, V)** represent tubers harvested from 4-month-old plants in greenhouse. Row 5: **(E, K, Q, W)**; row 6: **(F, L, R, X)** represent lateral and vertical sections of representative “medium-sized” tubers, which are stained with Lugol’s solution and harvested from 4-month-old plants in greenhouse, respectively. The black bar in row 5 indicates 1 cm. For list of mutational events, see **Table 2**.

**Figure 6:**
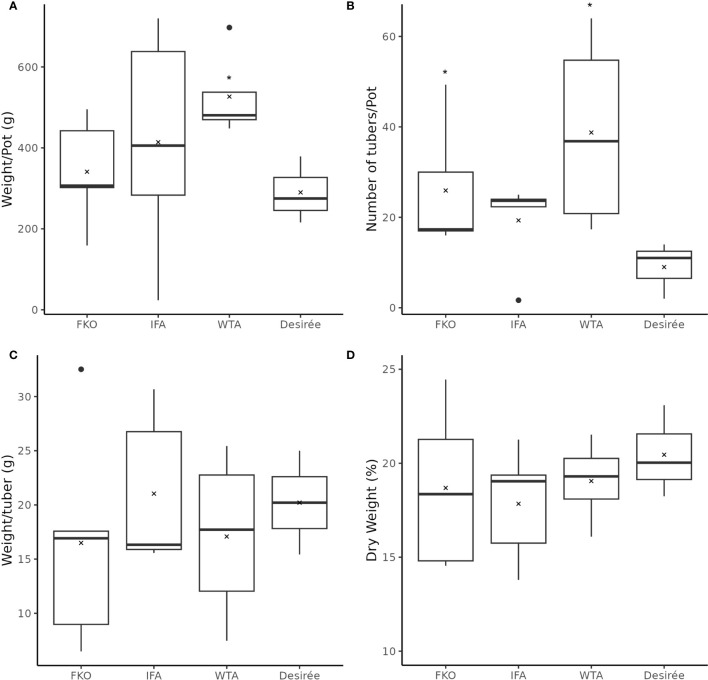
Phenotypic data of mutational events from greenhouse harvest at time of maturity. The above charts summarize four tuber characteristics of mutational events and Desirée (control). The mutational events are grouped according to groups, i.e., (from left to right) Group 1 (full knockouts; FKO), Group 2 (in-frame knockouts; IFM), and Group 3 (partial knockouts; WTA), and control. **(A)** Average total weight of tubers per plant (g), **(B)** average number of tubers per plant, **(C)** average weight per tuber (total weight of all tubers per pot/total number of tubers per pot; (g)), and **(D)** Average dry weight of tubers (percentage). All data were recorded in triplicates. Average and median values are represented as “X” and horizontal black bars. (*p<0.05, t-test—one tailed, two samples, equal variance).

The general phenotypic impact of induced mutations was investigated in greenhouse cultivation. In general, the plant height and growth rate of mutated events was similar to Desirée (control) during cultivation in greenhouse, except for SPD-9, which had an aberrant phenotype (**Figure 5**; **Supplementary Figure S4**). The average total weight of harvested tubers per pot was higher in all groups as compared to control (Desriee), however, it was only significant in WTA group (**Figure 6A**). Most events had two- to four fold higher number of tubers as compared to Desirée, except for SPD-9, which only produced two relatively small tubers/pot (**Figure 6B**; **Supplementary Figure S4**). SPD-1, SPD-5, SPD-11, and SPD-14 had 30, 49, 52, and 64 tubers/pot on average as compared to 9 tubers/pot for Desirée (**Figures 5**, **6B**; **Supplementary Figure S4**). Tubers from the majority of the events in the FKO and IFA groups were elongated, whereas tubers from the WTA group were more similar to Desirée (**Figure 5**; **Supplementary Figure S4**). SPD-5, SPD-11, and SPD-14 had a disproportionately high number of small round tubers (**Figures 5**, **6B**; **Supplementary Figure S4**). Overall, in most events, the number of tubers were higher than Desirée, and a majority of them (30%–60%) were smaller in size (**Figure 6B**; **Supplementary Figure S4**). The average weight per tuber (FW) was lower in FKO and WTA groups as compared to Desirée, except for IFA group, which was similar to Desirée (**Figure 6C**). However, the average dry matter content of the harvested tubers was mostly similar to Desirée (**Figure 6D**).

### 3.5 Full knockouts and in-frame mutants are deficient in Pho1a activity

**Figure 7:**
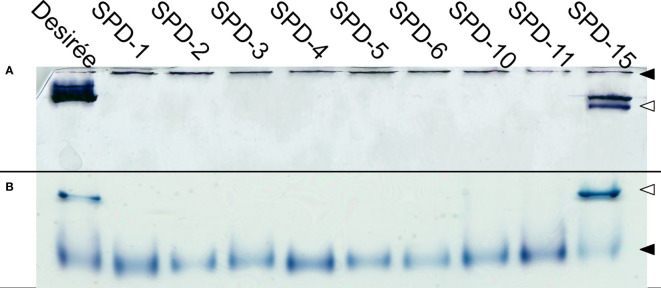
Phosphorylase activity gel. Protein extracts prepared from tubers of *Solanum tuberosum* were run on both native PAGE containing 0.8% glycogen **(A)**, and commercially available native PAGE **(B)**. Both conditions show clear activity for Pho2 (◀) for all the events, while only Desirée and SPD-15 show activity for Pho1a (◁).

To verify that the FKO events were deficient in Pho1a activity and to assess the other mutant groups, Zymograms were run on representatives of all three groups (**Figure 7**). Two types of zymograms were used, one glycogen containing running gel providing affinity retardation and primer (**Figure 7A**) and one lacking any added primer in the form of maltooligosaccharides, glycogen, or soluble starch (**Figure 7B**). Starch phosphorylase activity can be detected in both gels for Pho1a and the cytosolic form, Pho2. The migration of Pho2 is greatly affected by glycogen in the gel and can be found close to the well in **Figure 7A** but represents the fastest migrating activity band in **Figure 7B**. As expected, none of the events in the FKO group showed detectable activity for Pho1a. Interestingly, the same was true for all events with in-frame deletions (SPD-6 and SPD-10) and one of the mutants still carrying at least one wild-type alleles (SPD-11). This means that in-frame mutations where one or more amino acids are lost at the target site were detrimental for Pho1a activity. The only mutant with detectable Pho1a activity was SPD-15, which showed activity comparable to that of Desirée. This could indicate that all eight alleles encoding Pho1a do not have the same influence on total Pho1a activity.

### 3.6 Tuber amyloplasts of mutated events accumulate more starch granules

**Figure 8:**
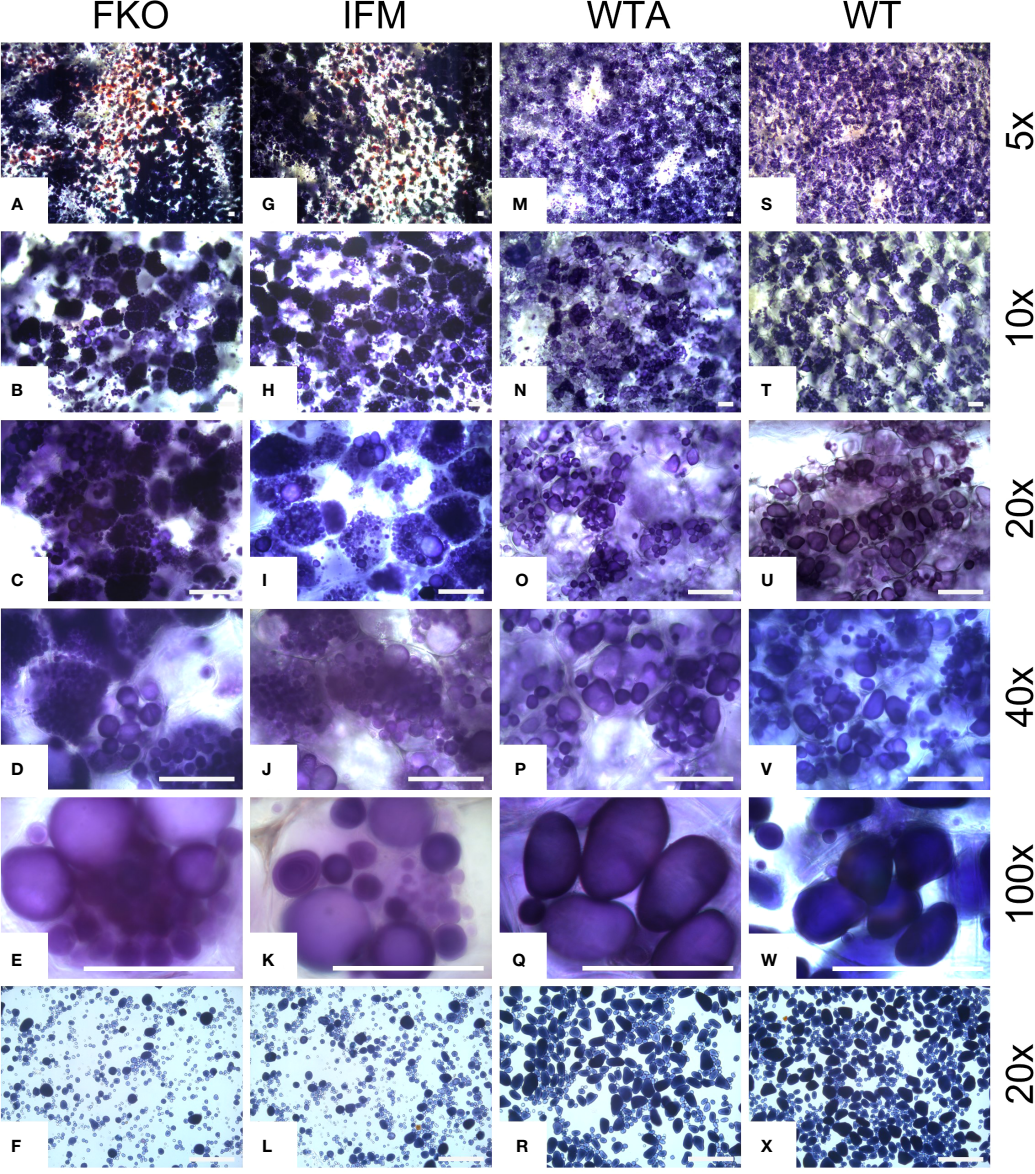
Light micrographs of starch granules stained with Lugol’s solution in tubers of representative mutational events. **(A–F)** SPD-1: Group 1 (full knockouts; FKO); **(G–L)** SPD-6: Group 2 (in-frame knockouts; IFM); **(M–R)** SPD-15: Group 3 (partial knockouts; WTA) and **(S–X)** Desirée (WT, control). Rows 1–5 represent stained tuber slices visualized under a microscope in increasing order of magnification under magnification as indicated in the figure. Each row has the same magnification, and black/white bar indicates 100 µm. For list of mutational events, see **Table 2**.

**Figure 9:**
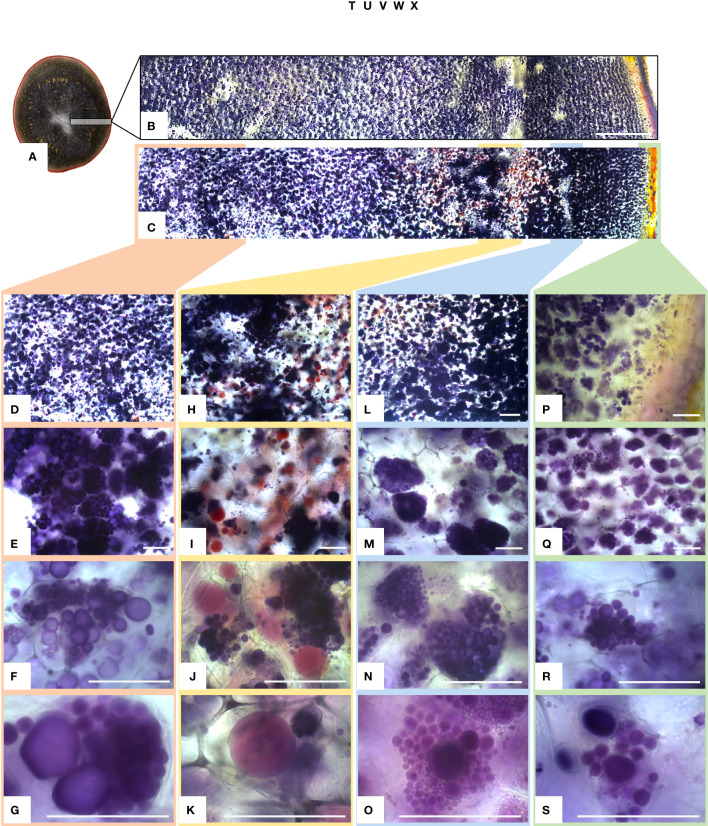
Light micrographs of starch granules stained with Lugol’s solution in tubers of mutational event SPD-1 from Group 1 (full knockouts; FKO) and Desirée control. **(A)** Lateral section of tuber stained with Lugol’s solution, harvested from 4-month-old plants in greenhouse. **(B, C)** Desirée and SPD-1, respectively, section of the tuber slice used for visualization under a microscope; left to right: center to periphery (skin) of tuber. Panels **(B, C)** are composite image of six individual images. Columns 1–4 (left to right: center to periphery (skin) of tuber slice) represent four areas under visualization with increasing magnification (top to bottom). **(D–G)** Center; **(H–K)** area around the vascular tissue; **(I–O):** area right to vascular tissue and **(P–S):**: periphery (skin) of tuber slice. Magnification is indicated in the figure. Each row has the same magnification, and black/white bar indicates 100 µm. For list of mutational events, see **Table 2**.

The amyloplasts from tubers of mutated events from both FKO and IFM groups contained an increased number of small granules as compared to Desirée (control) (**Figure 8**). The number of starch granules in WTA events were similar to Desirée (**Figure 8**). In general, amyloplasts from tubers of mutated events from both FKO and IFM groups predominantly contained a higher proportion of small granules (up to 80%), whereas the proportion of small granules in WTA group and Desirée varied from 30% to 60% (**Figure 8**). Smaller granules were found to be present in the amyloplast throughout the tubers from FKO and IFM groups, whereas for WTA and Desirée, the smaller granules were predominantly located in amyloplasts near the skin (**Figure 9**). The shape of the granules was more spherical in most FKO and IFM mutational events, irrespective of the size as compared to WTA events, which accumulated more oval granules similar to Desirée (**Figure 8**). Tubers from both FKO and IFM mutational events contained a high presence of reddish-brown stained spherical particles, in and around the vascular tissue of the tubers, which were rarely found in WTA events and Desirée (**Figures 8**, **9**). The number of starch granules per chloroplast in stomatal guard cells from leaf samples from all groups of mutated events were found to be comparable to Desirée (**Supplementary Figure 5**).

### 3.7 Full knock out of *Pho1a* affect the tuber starch content and amylose to amylopectin ratio is significantly decreased in tubers

**Figure 10:**
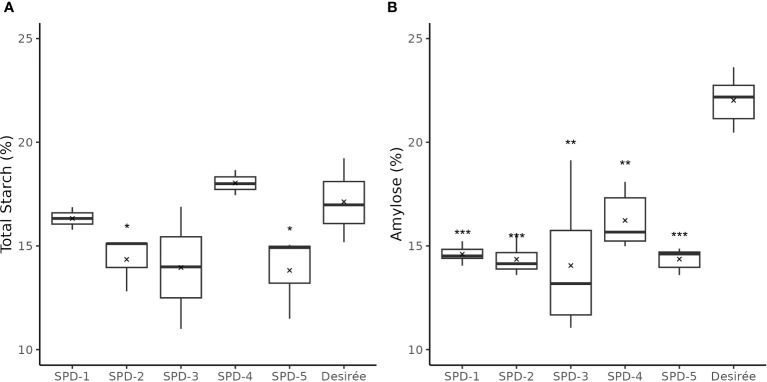
Total starch and amylose content in tubers of five full knockout events from Group 1 (FKO) and Desirée (WT, control). **(A)** Total starch measurements and **(B)** amylose fraction, represented as percentage of dry weight. All measurements were made in triplicates. Average and median values are represented as “X” and horizontal black bars. (*p<0.05, **p<0.01, ***p<0.001; *t-test—*one tailed, two samples, equal variance).

The total starch content in four of the five FKO events was found slightly lower as compared to Desirée (control) (**Figure 10A**). SPD-1, 2, 3, and 5 had an average of total starch content at 16.3%, 14.4%, 14.0%, and 13.8%, respectively, as compared to 17.1% for Desirée (DW), but only the decreased content in SPD-2 and 5 was found to be significant (**Figure 10A**). SPD-4 had a slightly, but not significant, higher average total starch content measuring 18.0% (DW) (**Figure 10A**).

The amylose content was found to be consistently lower in the FKO events at 14.1%–14.6% for SPD-1, 2, 3, and 5, and 16.2% in SPD-4, as compared to 22.0% in Desirée (DW) (**Figure 10B**; **Supplementary Figure S6**). Similarly, amylose content in IFM and WTA group was also decreased as compared to Desirée (**Supplementary Figure S6**). The amylose content in SPD-10 and SPD-11 samples at 16.4% and 15.4%, respectively, was comparable to FKO group, whereas the determined amylose content in SPD-6 and SPD-15 at 21.2% and 21.6% was more comparable to Desirée (**Supplementary Figure S6**).

As *StGBSS1* is responsible for amylose synthesis in potato tubers, the expression of the corresponding gene was investigated in the mutational events. The expression of *StGBSS1* was found to be lower in all mutational events (**Supplementary Figure S7**). The decrease in expression could possibly explain the observed decrease in amylose content, although there was not a consistent pattern. Overall, the most reduction in the expression levels of *StGBSS1* was observed in WTA group, followed by IFA and least in FKO group (**Supplementary Figure S7**).

### 3.8 *Pho1a* knockout does not prevent cold sweetening of tubers

**Figure 11:**
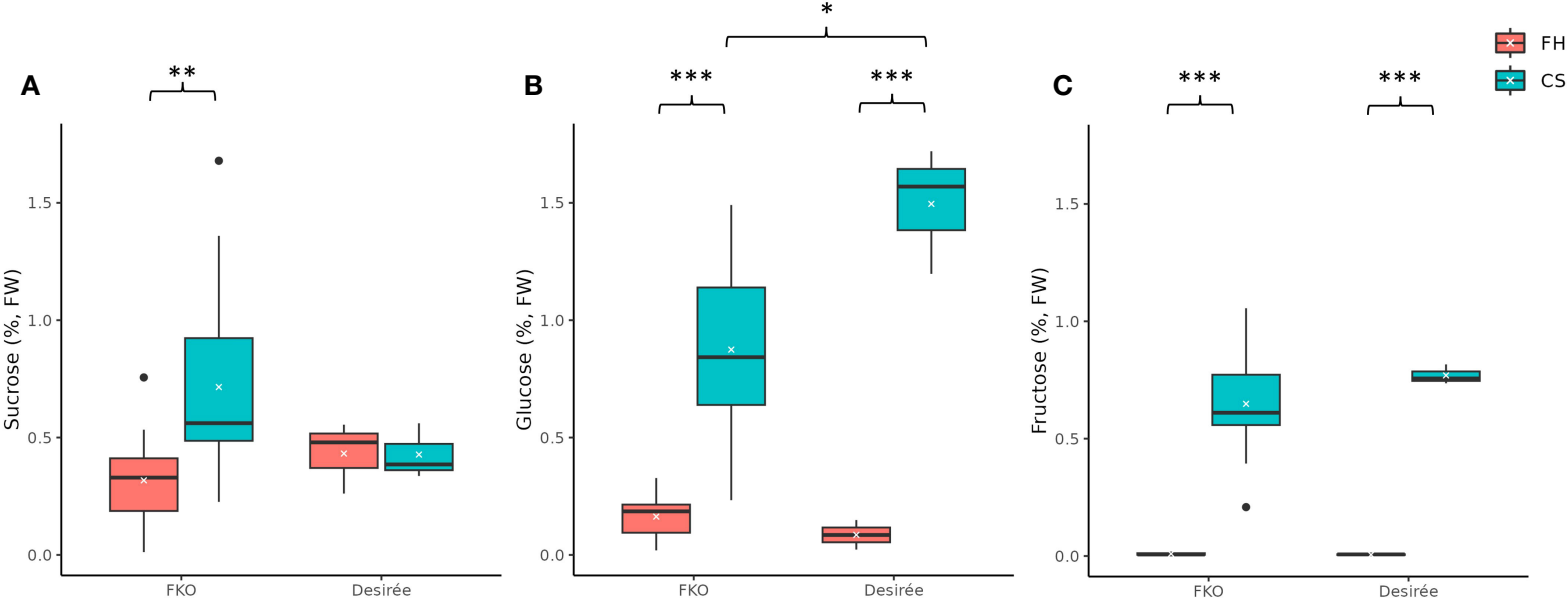
Free sugar levels in fresh and cold-stored tubers of five full knockout events from Group 1 (FKO) and Desirée (WT, control). **(A)** Sucrose, **(B)** glucose, and **(C)** fructose levels, represented as percentage of dry weight on respective y-axis. Fresh and cold-stored tuber samples are colored as orange and green, respectively. All measurements were recorded as triplicates. Statistical analyses were made on freshly harvested tubers and cold stored tubers, respectively. Average and median values are represented as “X” and horizontal black bars. (*p<0.05, **p<0.01, ***p<0.001; t-test—one tailed, two samples, equal variance).

Free sugar levels, i.e., concentration of sucrose, glucose, and fructose in potato tubers were measured at harvest and after a 3-month cold storage at 4°C in potato tubers of FKO mutational events (**Figures 11A–C**). In general, the levels of free sugars were higher in cold stored tubers for FKO events and Desirée (control).

The average free sucrose levels in cold stored tubers of FKO events was higher as compared to fresh tubers, differently from Desirée (control) where no significant change was detected in sucrose content between fresh and cold stored tubers (**Figure 11A**; **Supplementary Figure S8A**). The average free sucrose level of fresh tubers of the FKO group was slightly lower (0.32%) as compared to Desirée (0.43%) that increased in cold-stored FKO tubers (0.72%) as compared to no change in Desirée (0.43%) (**Figure 11A**; **Supplementary Figure S8A**). Very little to no free glucose was detected in fresh tubers of FKO mutational events (0.08%) and Desirée (0.09%) (**Figure 11B**; **Supplementary Figure 8B**), with significant increase in the cold-stored tuber samples. The average free glucose level was increased to 0.87% in tubers of the FKO group as compared to 1.50% in Desirée (**Figure 11B**). Similarly, the free fructose level in the fresh tuber samples of the FKO group and Desirée (control) was at the limit of detection but was significantly increased in respective cold-stored samples (**Figure 11C**). The average fructose content increased to 0.65% in FKO group tubers as compared to 0.77% in Desirée.

The higher average sucrose level of cold-stored FKO group tubers compared to Desirée (control), concomitant with a lower glucose level of cold-stored FKO group tubers compared to Desirée (control), could indicate that Pho1a has a role in cold sweetening of potato tubers.

## 4 Discussion

The role of plastid starch phosphorylase is still under investigation in plants. Even though it is one of the most highly expressed genes in potato tubers and one of the most prominent enzymatic activities in amyloplasts, its role, if any, for reserve starch synthesis is still unclear. In this manuscript, we identified that the major form, Pho1a, is encoded by a duplicated gene locus, and we further characterized its role for reserve starch composition and phenotype by observing CRISPR/Cas9 mutants of *Pho1a*.

The first indication of a duplicated *Pho1a* locus was found by the detection of up to seven differently mutated alleles in a single regenerated potato event. A plausible cause for this could be chimeric regeneration consisting of differently mutated somatic cells. However, applying RNP CRISPR/Cas9 tools to potato protoplasts generally avoids this problem from the transient nature of the application. Stable transformation for CRISPR/Cas9 mutations carries a higher risk of creating chimeric plants as mutations can occur at any time during the regenerative and vegetative phase. Copy number estimation of the *Pho1a* locus by RT-qPCR confirmed that more than one copy exists in the parental variety Desirée. A duplication of the *Pho1a* locus was further verified, analyzing the current available potato genome assembly DM v6.1. In addition, duplication of the *Pho1a* locus was also evident in multiple assembled wild potato genomes. The *Pho1a* copies on chromosome 3 were found to have highly conserved sequences in the DM v6.1 assembly. A ~7.5-kbp upstream region of both copies was also highly conserved. With the exception of a ~1.3-kbp long upstream genomic segment, ~25 kbp long genomic segment harboring *Pho1.1a* and ~23 kbp long genomic segment encompassing *Pho1.2a* were nearly identical. Both segments were delimited by ~300-bp-long conserved sequences, which were only detected in *Pho1a* loci from cultivated tetraploid potato cultivars. The presence of these conserved sequences may act as recombination hotspots during meiosis.

A reference mRNA sequence of *Pho1a* (X52385.1) spanned across 15 exons that were highly conserved in wild and cultivated potato assemblies, indicating an evolutionary constraint. However, the intronic regions were less conserved and contained species specific insertions. As a result, the length of a putative *Pho1a* locus ranged from 8,440 to 16,228 bp, depending on insertion and length of *Tst1* in intron 5 and species-specific insertions in intron 2, 9, 11, and 13.

The duplicated *Pho1a* locus is miss-annotated in the DM v6.1 assembly where *Pho1.1a* locus is annotated as Soltu.DM.03G007710 and Soltu.DM.03G007720. Both genes share a high sequence identity with exons 1–5 and exons 6–15 of reference mRNA sequence of *Pho1a.* Similarly, *Pho1.2a* locus is annotated as Soltu.DM.03G007750 and Soltu.DM.03G007760, which share high sequence identity to exons 1 and 2, 3–5 and exons 6–15 of reference mRNA sequence of *Pho1a*, respectively. A comparison of putative *Pho1a* loci among DM v6.1, DM v8.1, DM1S1, and *S. tuberosum* Group Phureja assemblies of E86-69 and E4-63 indicated probable sequencing errors leading to putative stop codons and thus resulting in mis-prediction of coding sequences of above genes. Furthermore, these sequencing errors are resolved in DM1S1, and duplicated *Pho1a* loci are annotated as Soltu.DM1S1.03G006810, i.e., *Pho1.1a* and Soltu.DM1S1.03G006830, i.e., *Pho1.2a.*

*Pho1a* has previously been reported to contain the transposon sequence *Tst1* in the intron between exons 5 and 6 (CamIrand et al., 1990). It could be speculated that the transposon insertion could have triggered a gene duplication. However, *Tst1* sequences were detected in both single and duplicated *Pho1a* genes across 24 wild potato species. The *Tst1* sequences were found to be highly variable among species; however, both *Tst1* sequences of duplicated copies were nearly identical. Insertion of *Tst1* was not detected in *Pho1a* sequences from *S. palustre* and *S. etuberosum* from *Etuberosum* section or tomato, and coding sequence of *Pho1a* from tomato spanned only 14 exons. This indicates separate origins of found duplications of *Pho1a* and independence of *Tst1* presence. The duplication of *Pho1a* and insertion of *Tst1* must have originated in distinct duplication events during evolution. In addition, *Pho1b* genomic sequence was found to span 14 exons similar to *Pho1* from Etuberosum section and tomato, lacking *Tst1* insertion, which may suggest that *Pho1b* is the ancestral copy of *Pho1* gene.

Tuber development of complete knockout events was clearly affected. Tubers were elongated, smaller, and the number per plant was higher than the control. This may suggest that Pho1a has an important role in sink development and maintenance, affecting tuber setting and growth. An altered tuber sink capacity might result in the induction of more tubers from available sucrose transport. The overall tuber weight, dry matter, and starch content were similar to the control, which shows that total sink capacity into tubers and starch accumulation is not affected. On the other hand, the amylose content of the mutational events was significantly reduced in tubers of mutational events from the FKO group, although the reduction in amylose among IFM and WTA groups was more variable. This observation may have different explanations, since it is apparent amylose content that is measured. Thus, structural changes could either be in long chains of amylopectin or in amylose. StGBSS1 (granule bound starch synthase) is the enzyme responsible for amylose synthesis, and the expression of the corresponding gene was assessed. The relative expression of *StGBSS1* was found to be reduced in all mutational events. Interestingly, the reduction in the expression was highest in mutational events from WTA, followed by IFM and FKO groups. Taken together, this suggests a complex interaction of expression regulation among starch biosynthetic genes and possible protein–protein interactions, which needs to be further elucidated in potato.

Starch phosphorylase activity of mutant events was investigated via banding patterns in native PAGE gel zymograms with and without glycogen in the separation gel. Glycogen functions as affinity retardation for starch phosphorylase proteins and a possible primer for their biosynthetic activity. No Pho1a activity was detected in mutational events from FKO and IFM groups, which shows that full knockout and in-frame induced mutations lead to complete lack of Pho1a activity in respective events. However, Pho1a activity was only detected in SPD-15 and not in SPD-11 from WTA group. For the IFM group, this indicates that the amino acids not present in the mutant allele are crucial for detectable activity or that the particular in-frame mutant allele is of lesser importance as an allele for Pho1a activity. The results of the WTA group may suggest that there is an allelic variance among the eight alleles of *Pho1.1a* and *Pho1.2a* regarding contribution to Pho1a activity. Consistent with the literature (Albrecht et al., 1998), the Pho2 activity band migration was greatly retarded in the glycogen containing gel while having greater migration than Pho1a in a non-glycogen gel. Pho1a and Pho2 was observed to yield activity bands without a deliberately added primer in the form of maltooligosaccharides, glycogen, or starch.

While the AGPase-mediated starch biosynthesis is considered to be the absolute major pathway for reserve starch, plastid starch phosphorylase has been suggested to play a role for reserve starch accumulation, at least in some species and under some conditions (Hwang et al., 2020). A reduction in the size of the starch granules and a change in amylopectin chain length distribution due to mutations of *Pho1* has been reported in rice endosperm (Satoh et al., 2008). Similarly, the amyloplasts in the tubers from our mutated events were found to contain a large number of small starch granules. The abundance of small granules was higher at the periphery of the tuber tissue. Larger mature granules were also found, but then mostly located towards the center of the tubers. In addition, the shape of the granules was consistently rounder irrespective of the size as compared to the control. The roundish structure could be due to changed chain distribution or growth direction of starch and hence granule formation. Plastid starch phosphorylase could be of importance for the synthesis of long chains in amylopectin that might be reflected in the observed decrease in apparent amylose content. A change in chain length distribution where iodine no longer detects certain chains as amylose because of them now being shorter.

The accumulation of massive number of small granules may come from impairment in a biosynthetic and degradative direction. Pho1 has been suggested to be involved in the degradation of MOs chains to G-1-Ps in rice, where the MOs are resulting from trimming of pre-amylopectin by isoamylase-type DBEs (Hwang et al., 2010; Hwang et al., 2016a; Hwang et al., 2020). In wheat, Pho1 has been suggested to act phosphorolytically, directly at the surface of the granule (Tickle et al., 2009) and on linear glucans originating from degradation of starch granules (Tetlow and Bertoft, 2020). For both routes, this would release G-1-P, which converted to ADP-glucose, and might be utilized by SSs for transfer to other growing granules (Mérida and Fettke, 2021). In this way, Pho1a may contribute, together with other enzymatic activities, to ensure building, trimming, and structuring of the final starch granule phenotype and indirectly keep the number of granules in amyloplasts down.

A proliferation of smaller non-staining granules and red-stained granules was detected in the amyloplasts surrounding vascular tissue of the tubers of most full knockout mutational events. A similar phenotype has been reported previously in the tubers of transgenic events with antisense RNA suppression of two isoamylase genes, i.e., *Stisa1* and *Stisa2* in cv Desiree (Bustos et al., 2004). These very small granules were reported to consist of both amylose and amylopectin; however, the ratio and molecular masses of both polymers and organization were reported to be substantially different from large granules. The differences were attributed to the small surface area for amylopectin synthesis, resulting in lack of growth rings. The red-stained granules, on the other hand, were reported to have a much higher proportion of short chains than potato amylopectin and were more similar to phytoglycogen from the *sugary*1 mutant of maize. Phytoglycogen molecules result in spherical structures due to intrinsic lack of granular organization arising from a high degree of branching with short α-1,4 glucan chains. However, in our study, the isoamylase activity was not targeted, and no reports of any multi-enzyme complexes involving Pho1a and DBEs exist so far (Zhong et al., 2022). Pho1b has been shown to be specifically located to vascular tissue in potato tubers while the corresponding gene predominantly being expressed in leaf tissue (Albrecht et al., 2001). This leads to the possibility that extraction of RNA from whole tuber sample may result in the misrepresentation of expression in vascular tuber tissue. Pho1b is reported to exist as a heterodimer complex with Pho1a in potato leaves (Albrecht et al., 1998; Albrecht et al., 2001). It could be hypothesized that proliferation of tiny and red-stained granules may be the result of loss of a heterodimer complex in amyloplasts surrounding vascular tissue, although this would need further experimentation.

Pho1 has been suggested to play a role in the transitory starch degradation due to high [Pi] concentration (Kruger and ap Rees, 1983). However, downregulation of *PHS1* in *Arabidopsis* (Zeeman et al., 2004) and *Pho1b (STP-1)* in potato leaves (Sonnewald et al., 1995) did not significantly alter starch structure or diurnal starch metabolism, suggesting that Pho1 is not a major determinant in starch metabolism under normal conditions (Hwang et al., 2020). In agreement with above, knockout of *Pho1a* did not alter the number or size of starch granules in leaves of mutational events.

Pho1a has been suggested to be involved in the regulation of potato response to changes in temperature; however, effects of cold stress and cold storage of tubers have opposite effects (Slugina et al., 2020a). While potato tubers grown at low temperature had no significant effect on tuber starch content and granule size (Orawetz et al., 2016), the tubers stored at low temperature resulted in decreased tuber starch and increased free sugar accumulation along with increased Pho1a activity (Schreiber et al., 2014; Slugina et al., 2020a). The free sugar content in cold stored tubers of the fully mutated events was found to be higher than for fresh tubers. However, the increase in free sucrose, glucose, and fructose followed different patterns in comparison to Desirée (control). While the average sucrose level in cold-stored tuber samples was higher, the glucose level was significantly lower and with fructose on a similar level to cold-stored tuber samples from control. Potato tubers remain metabolically active, and starch degradation and sucrose accumulation are a perquisite for loss of post-harvest ecodormancy (Sonnewald and Sonnewald, 2014). Exposure to cold storage is suggested to influence duration of dormancy by affecting activity levels of saccharolytic enzymes like sucrose synthase, or vacuolar acid invertase and its inhibitor (Slugina et al., 2020a). The concomitant higher sucrose and lower glucose level of the FKO group in comparison to the Desirée control indicate a role of Pho1a in cold sweetening of potato tubers.

## 5 Conclusion

*Pho1a* was shown to be tandemly duplicated in potato and thus named *Pho1.1a* and *Pho1.2a*. The gene duplication does not exist in tomato but can be found in species more related to *Solanum tuberosum*. Both genes encoding Pho1a were mutated using CRISPR/Cas9, and the impact on tuber development and starch accumulation was investigated in full knockouts. The results showed that lack of Pho1.1a and Pho1.2a have a large impact on tuber shape, size, and number per plant. Furthermore, an increase in small starch granules and more round granule shape was found, while the overall apparent amylose content was decreased. A differential, although low effect from absence of Pho1a, was observed upon cold storage of tubers that may indicate a role for Pho1a in starch degradation and cold sweetening. This study firmly establishes Pho1.1a and Pho1.2a as an important enzymatic factor in forming the starch granule phenotype and structure in reserve starch accumulation of potato tubers.

## Data availability statement

The original contributions presented in the study are included in the article/**Supplementary Files**, further inquiries can be directed to the corresponding author/s.

## Author contributions

PH conceptualized the study. PH, MA, and SS planned the study. SS, MF, MA, PV, HT, and NO performed experiments. All authors analyzed results. SS and PH wrote the manuscript. SS, PH, MA, and MF edited the manuscript. All authors approved the manuscript in its final form.
