# Supplementary material for: Pho1a (plastid starch phosphorylase) is duplicated and essential for normal starch granule phenotype in tubers of Solanum tuberosum L
Source: Front Plant Sci. 2023 Aug 9;14:1220973. doi: 10.3389/fpls.2023.1220973 (PMC10450146; doi:10.3389/fpls.2023.1220973)
Supplement: Supplementary file 1 [file DataSheet_1.pdf]

Supplementary Figure 1:

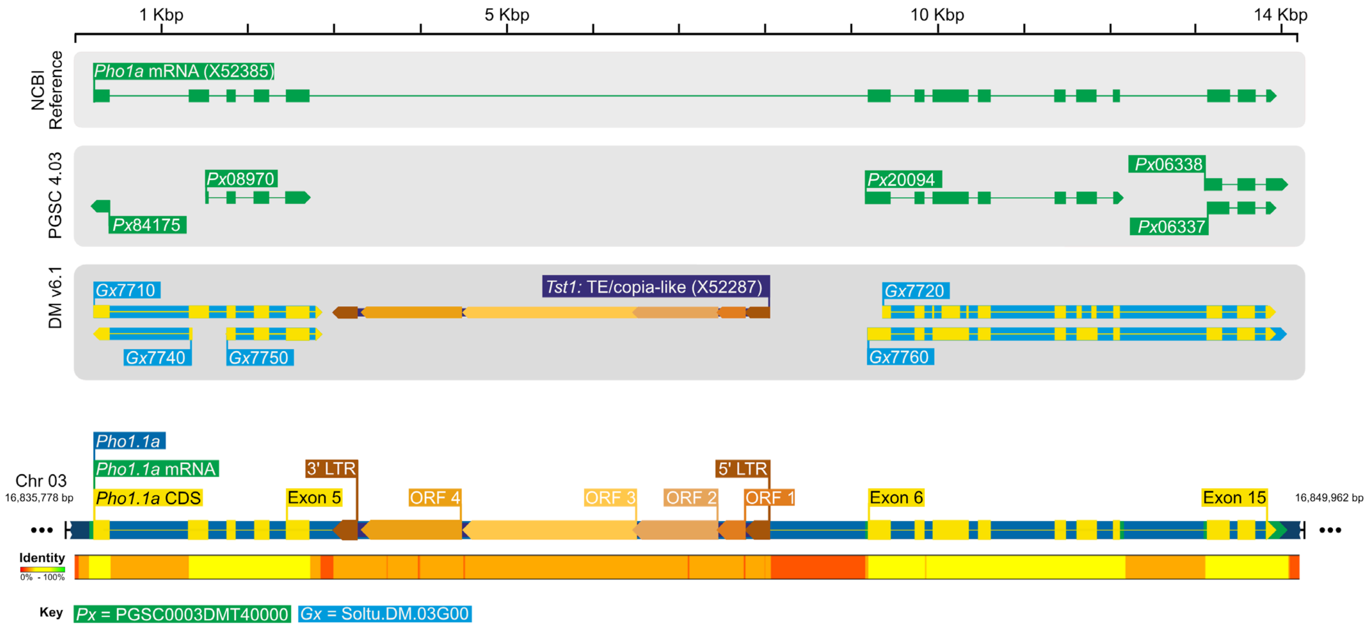

Alignment of *Pho1.1a* genomic *sequences from S. tuberosum* Group Phureja DM 1-3 516 R44 Genome Assembly (DM v6.1), previously reported *Pho1a* mRNA sequences from NCBI, PGSC v4.03 and TE/copia like retrotransposon *Tst1*. The respective sequence IDs are annotated and sequence identity conservation is represented as color coded legend at the bottom.
