# Supplementary material for: Pho1a (plastid starch phosphorylase) is duplicated and essential for normal starch granule phenotype in tubers of Solanum tuberosum L
Source: Front Plant Sci. 2023 Aug 9;14:1220973. doi: 10.3389/fpls.2023.1220973 (PMC10450146; doi:10.3389/fpls.2023.1220973)
Supplement: Supplementary file 2 [file DataSheet_2.pdf]

Supplementary Figure 2:

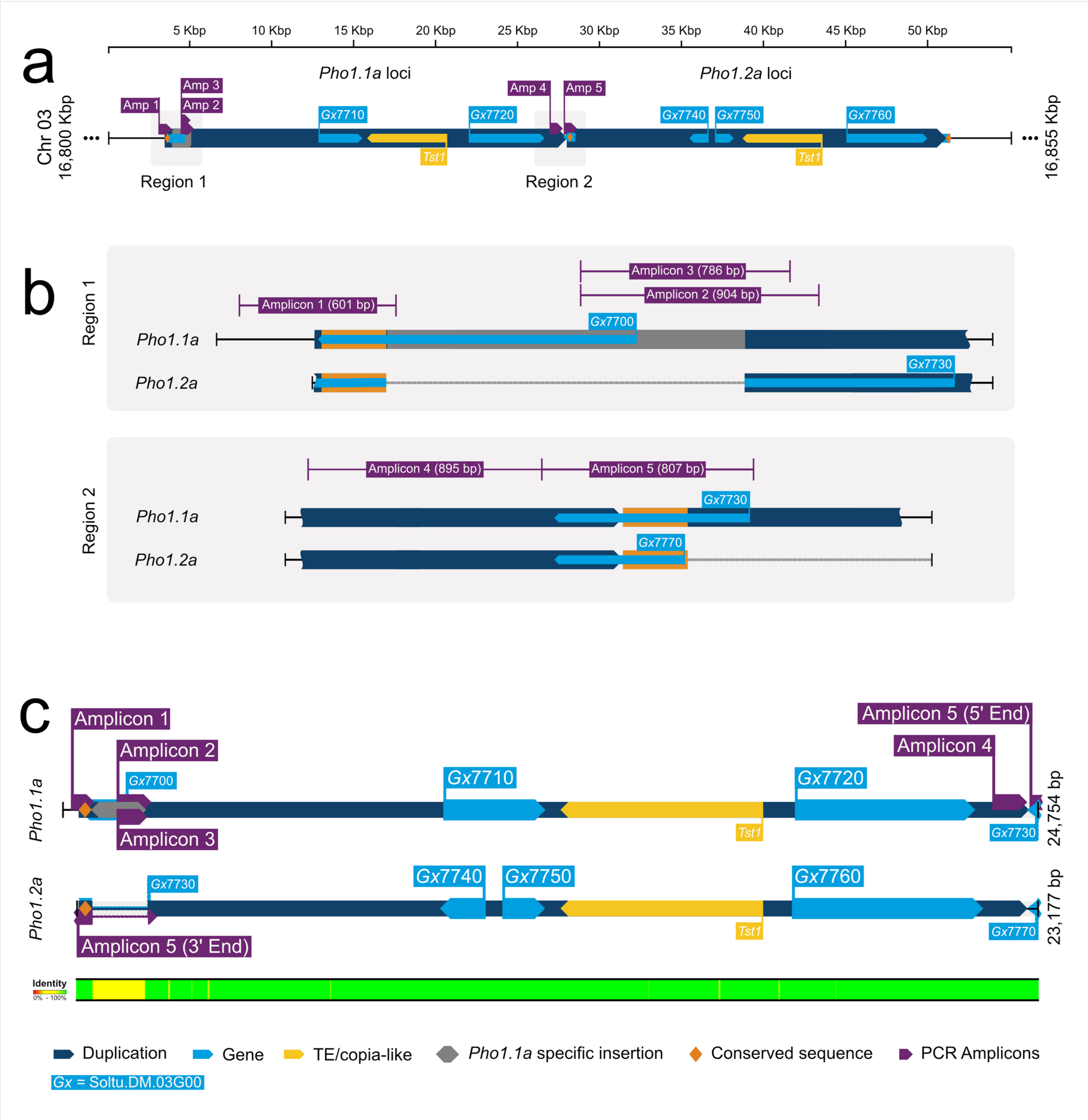

A graphical representation of duplicated *Pho1a* genomic loci in DM v6.1.
a) The duplicated genomic segments as dark blue blocks and gene annotations in DM v6.1 as light blue blocks. b) Enlarged view and sequence comparison of *Pho1.1a* and *Pho1.2a* loci in the highlighted regions as region 1 and 2 in 'a'. c) Sequence alignment of *Pho1.1a* and *Pho1.2a* loci. The targeted cloning and sequencing amplicons are in purple, *Pho1.1a* specific insertion sequence in grey and conserved repeating sequence as yellow. The respective sequence IDs are annotated and sequence identity conservation is represented as color coded legend at the bottom.
