# Supplementary material for: Pho1a (plastid starch phosphorylase) is duplicated and essential for normal starch granule phenotype in tubers of Solanum tuberosum L
Source: Front Plant Sci. 2023 Aug 9;14:1220973. doi: 10.3389/fpls.2023.1220973 (PMC10450146; doi:10.3389/fpls.2023.1220973)

Pho1a (plastid starch phosphorylase) is duplicated and essential for normal starch granule phenotype in tubers of *Solanum tuberosum* L.

Shrikant Sharma[1,*], Martin Friberg[1], Paul Vogel[1], Helle Turesson[1,2], Niklas Olsson[1], Mariette Andersson[1], Per Hofvander[1,*]

*** Correspondence:**

Shrikant Sharma
shrikant.sharma@slu.se

Per Hofvander
per.hofvander@slu.se

Supplementary Figure 3:

HFRA electrograms of *Pho1a* mutational events from Table 2.

HFRA Electrograms, Group 1: (Full knockouts; FKO)

Desirée (WT, cont.)

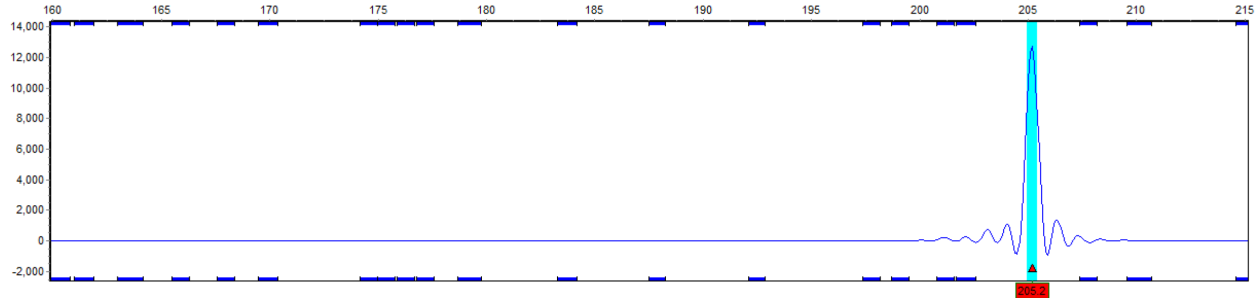

SP-Des-1 (181057)

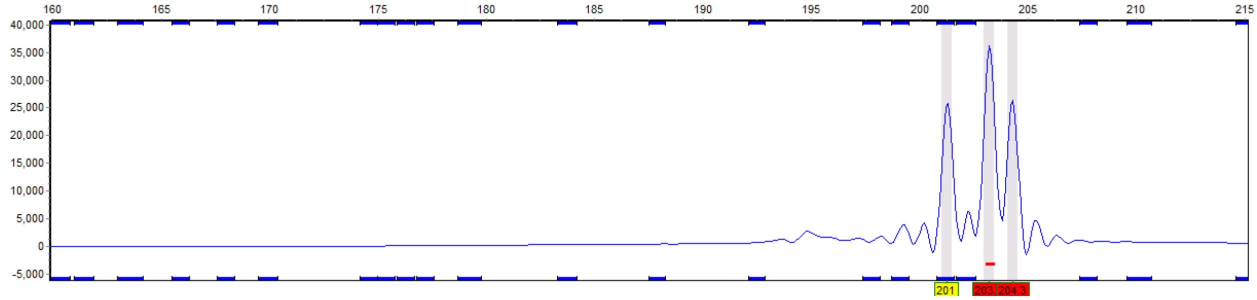

SP-Des-2 (181085)

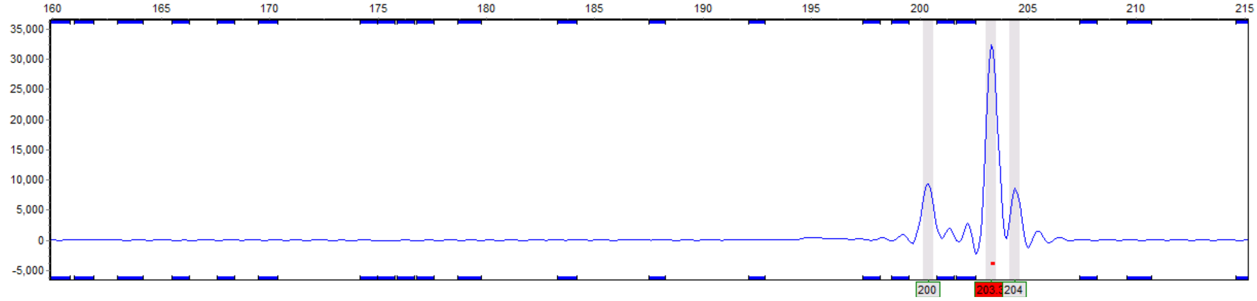

SP-Des-3 (181092)

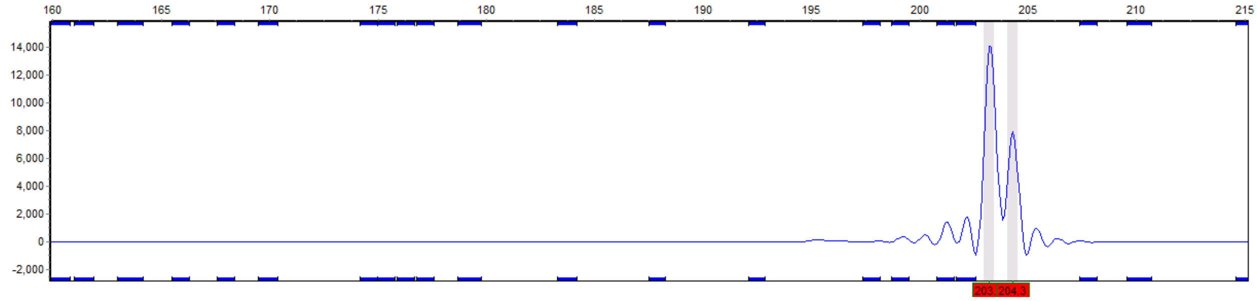

SP-Des-4 (181113)

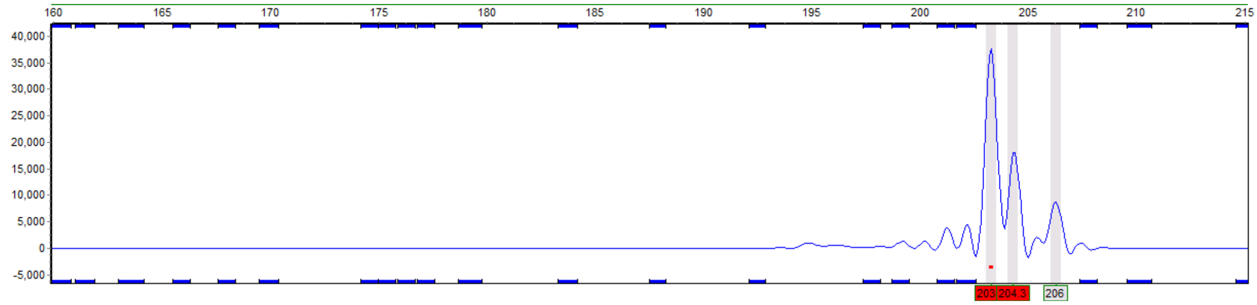

SP-Des-5 (181124)

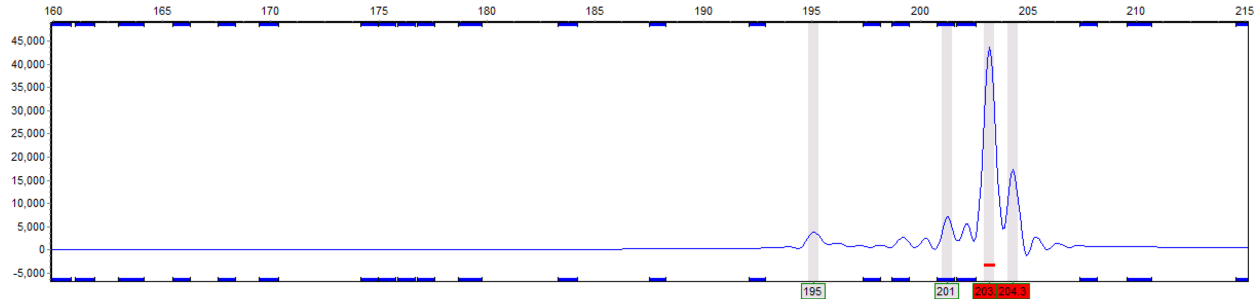

HFRA Electrograms, Group 2: (In-Frame Mutation; IFM)

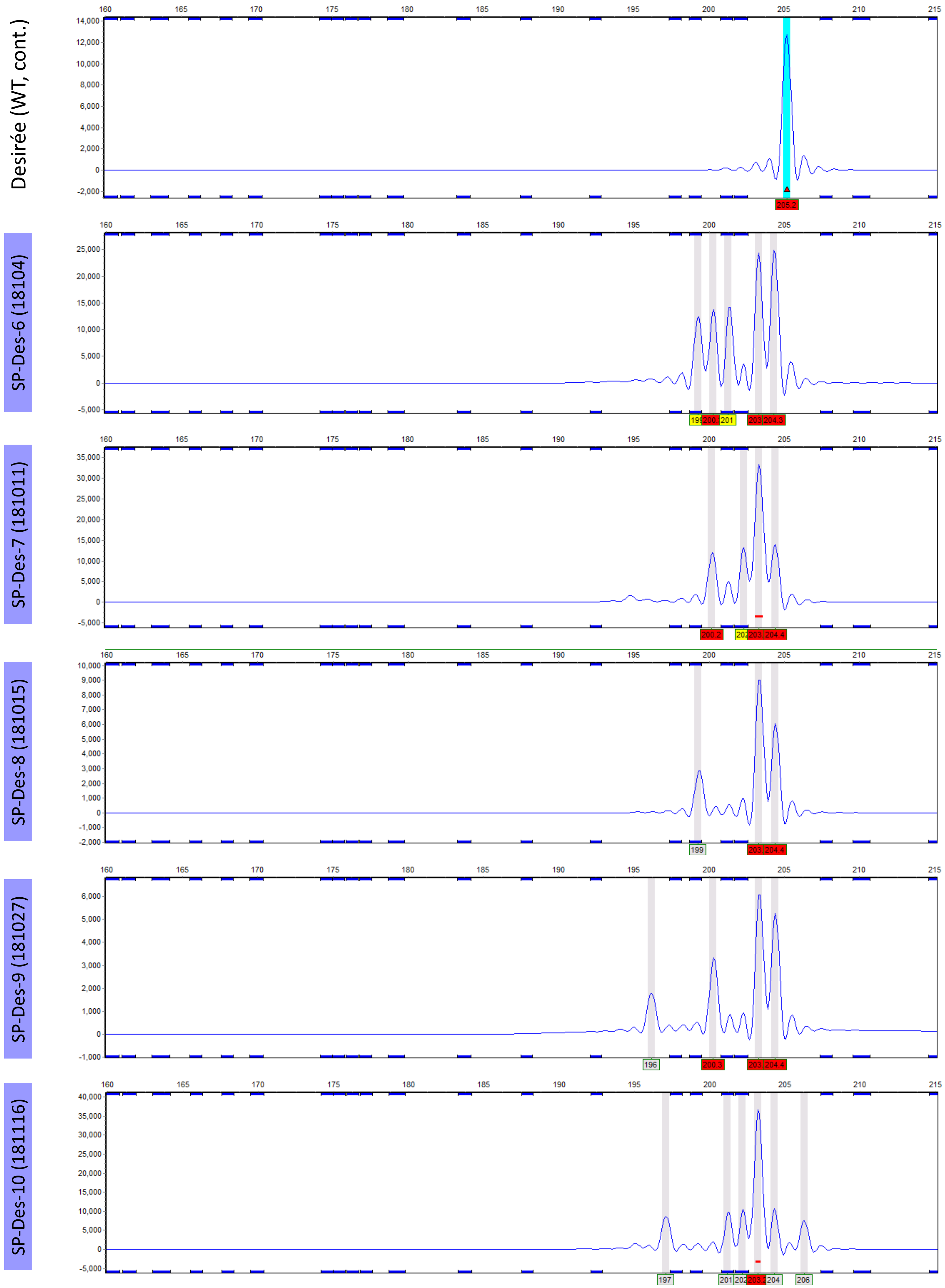

HFRA Electrograms, Group 3: (containing wild type alleles; WTA)

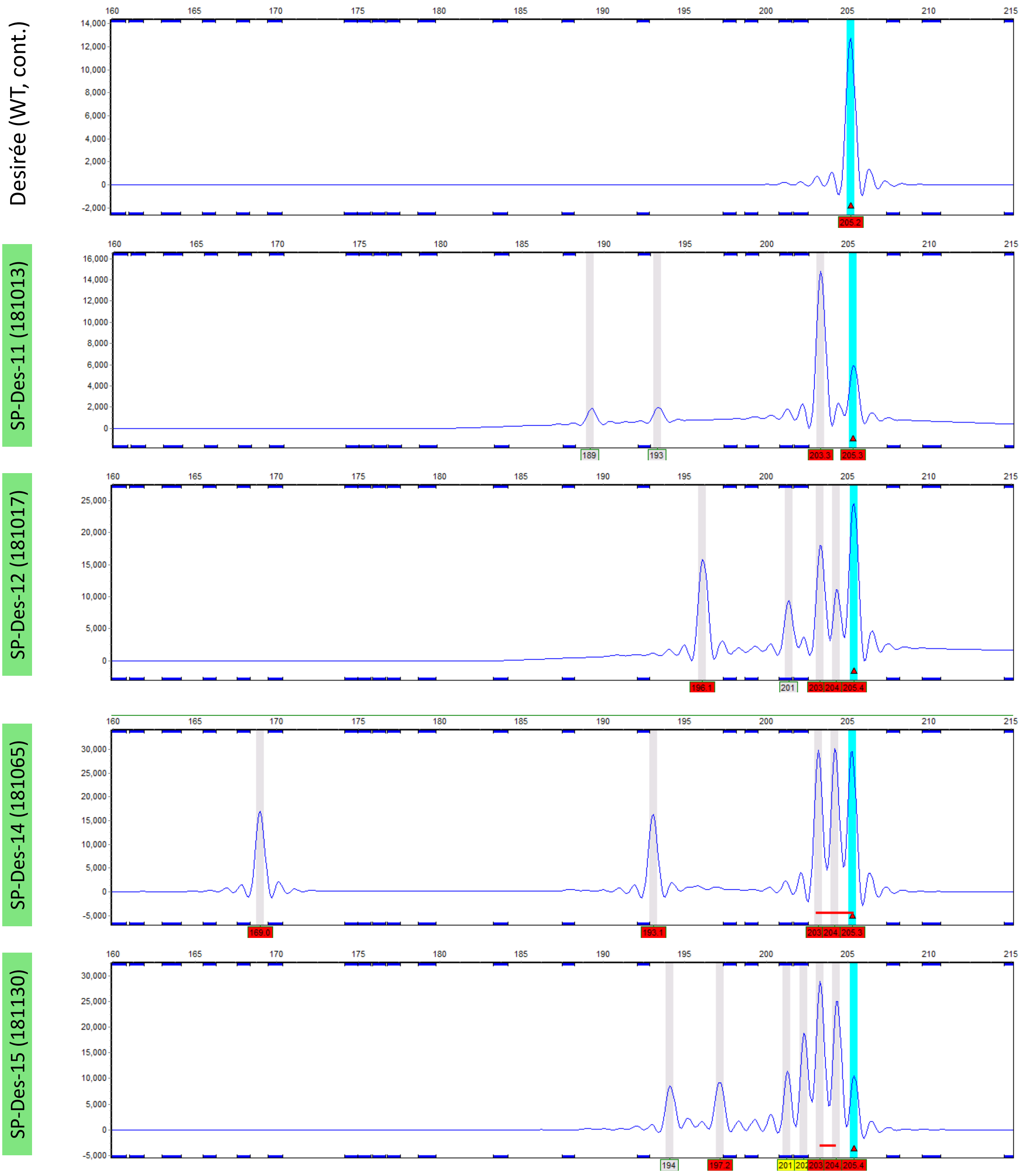

Supplement: Supplementary file 3 [file DataSheet_3.pdf]
