# Supplementary material for: Pho1a (plastid starch phosphorylase) is duplicated and essential for normal starch granule phenotype in tubers of Solanum tuberosum L
Source: Front Plant Sci. 2023 Aug 9;14:1220973. doi: 10.3389/fpls.2023.1220973 (PMC10450146; doi:10.3389/fpls.2023.1220973)

Pho1a (plastid starch phosphorylase) is duplicated and essential for normal starch granule phenotype in tubers of *Solanum tuberosum* L.

Shrikant Sharma[1,*], Martin Friberg[1], Paul Vogel[1], Helle Turesson[1,2], Niklas Olsson[1], Mariette Andersson[1], Per Hofvander[1,*]

*** Correspondence:**

Shrikant Sharma
shrikant.sharma@slu.se

Per Hofvander
per.hofvander@slu.se

Supplementary Figure 4:

Pictures of tubers from greenhouse cultivation and harvest of *Pho1a* mutational events from Table 2.

Tuber harvest, Group 1: (Full knockouts; FKO)

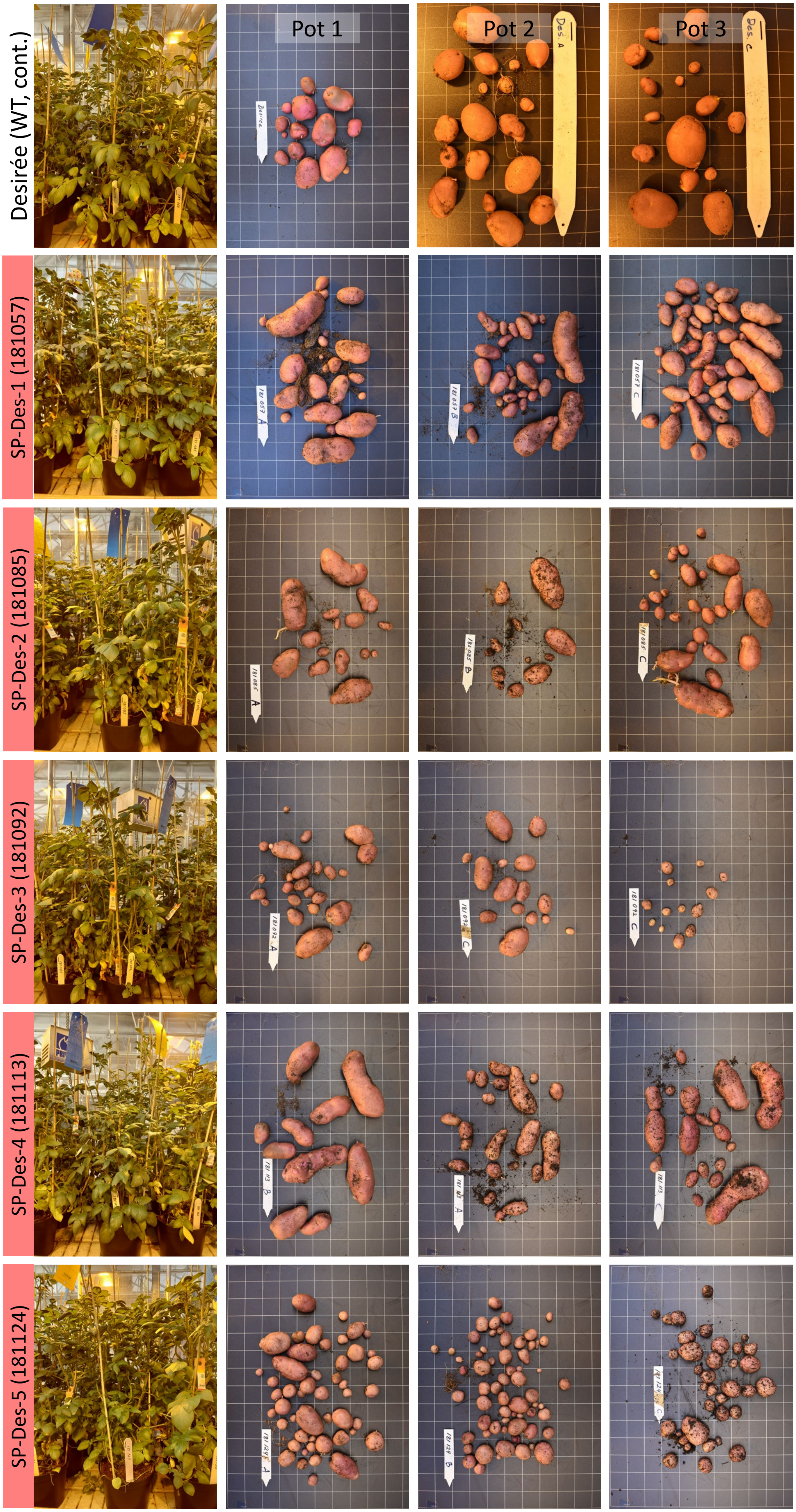

Tuber harvest, Group 2: (In-Frame Mutation; IFM)

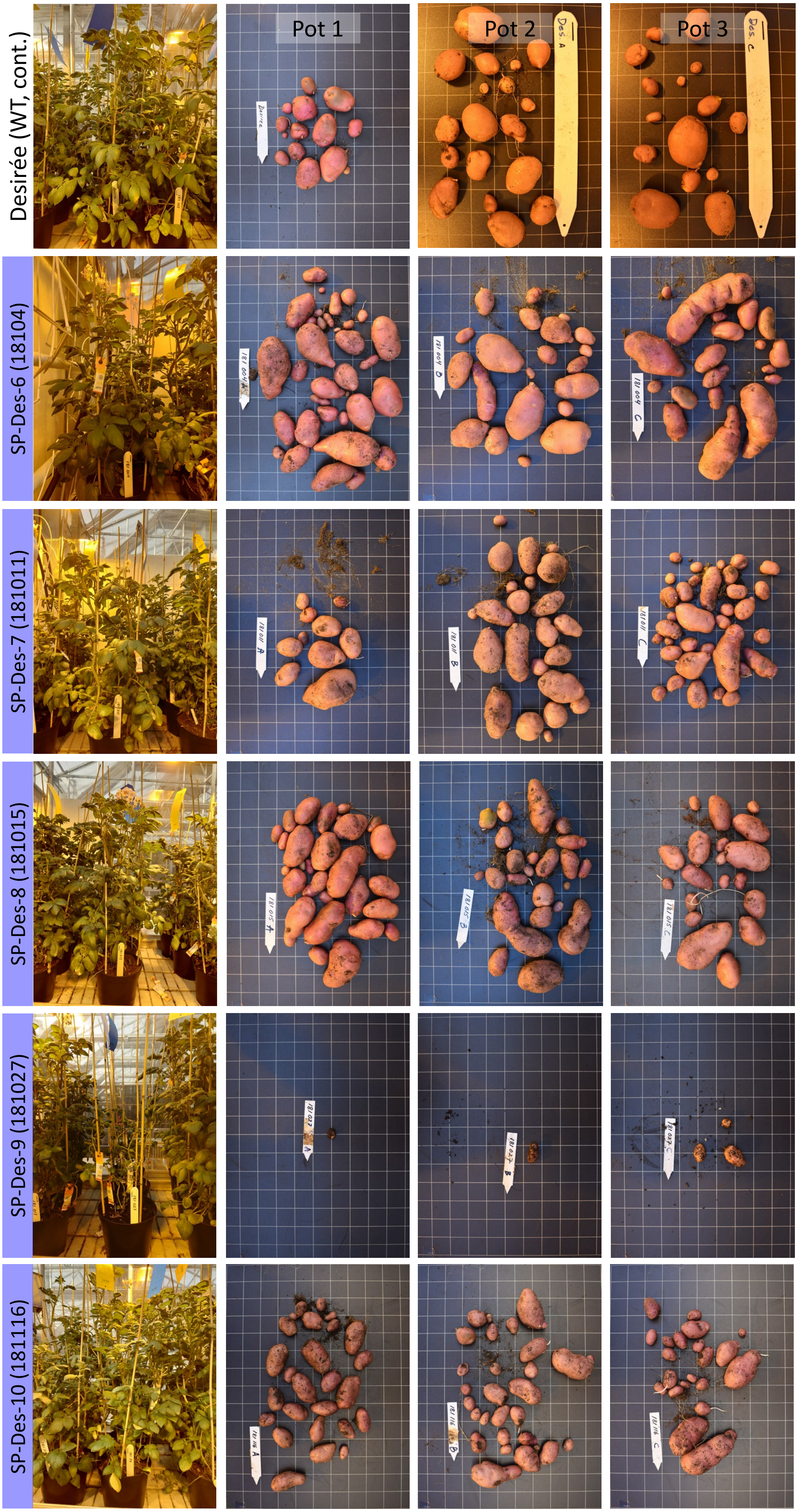

Tuber harvest, Group 3: (containing wild type alleles; WTA)

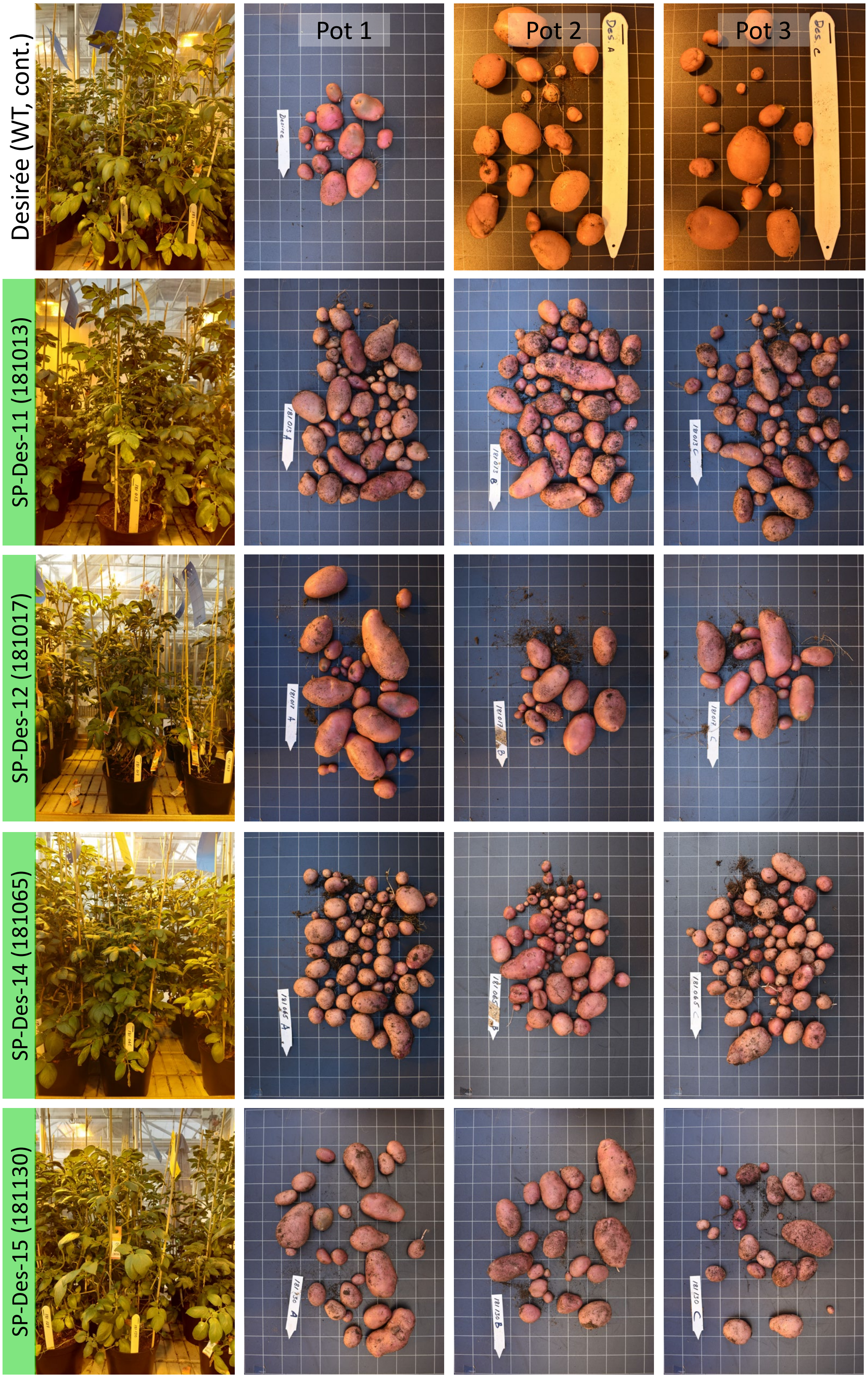

Supplement: Supplementary file 4 [file DataSheet_4.pdf]
