# Supplementary material for: Pho1a (plastid starch phosphorylase) is duplicated and essential for normal starch granule phenotype in tubers of Solanum tuberosum L
Source: Front Plant Sci. 2023 Aug 9;14:1220973. doi: 10.3389/fpls.2023.1220973 (PMC10450146; doi:10.3389/fpls.2023.1220973)
Supplement: Supplementary file 5 [file DataSheet_5.pdf]

# *Pho1a* (plastid starch phosphorylase) is duplicated and essential for normal starch granule phenotype in tubers of *Solanum tuberosum* L.

Shrikant Sharma[1,*], Martin Friberg[1], Paul Vogel[1], Helle Turesson[1,2], Niklas Olsson[1], Mariette Andersson[1], Per Hofvander[1,*]

*** Correspondence:**

Shrikant Sharma
shrikant.sharma@slu.se

Per Hofvander
per.hofvander@slu.se

## Supplementary Figure 5:

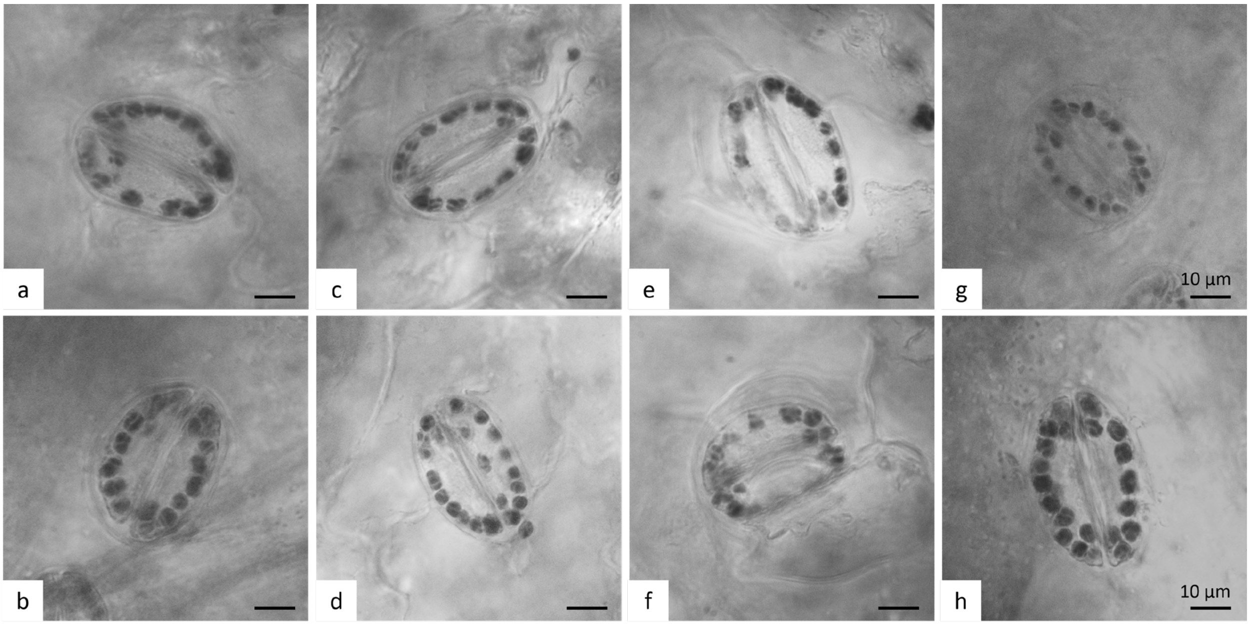


**Light micrographs of leaves segments of *Pho1a* mutational lines.**
One representative event from each group shown, i.e., from left: Column 1, a-b: SPD-1 (Full knockouts; FKO); Column 2, c-d: SPD-6 (In-Frame knockouts; IFM); Column 3, e-f: SP-D-15 (Partial knockouts; WTA) and Column 4, g-h: Desirée (WT, control). Row 1 and 2 represent Lugol's stained leaves depicting stomatal cells on dorsal surface visualized under microscope at 40X. First row: a, c, e and g are leaf samples harvested at the middle of the light phase and second row: b, d, f and h end of the light phase for respective lines. Dark dots indicate starch granules located in chloroplasts. The black bar indicates 10 µm.
