# Supplementary material for: Pho1a (plastid starch phosphorylase) is duplicated and essential for normal starch granule phenotype in tubers of Solanum tuberosum L
Source: Front Plant Sci. 2023 Aug 9;14:1220973. doi: 10.3389/fpls.2023.1220973 (PMC10450146; doi:10.3389/fpls.2023.1220973)
Supplement: Supplementary file 6 [file DataSheet_6.pdf]

Supplementary Figure 6:

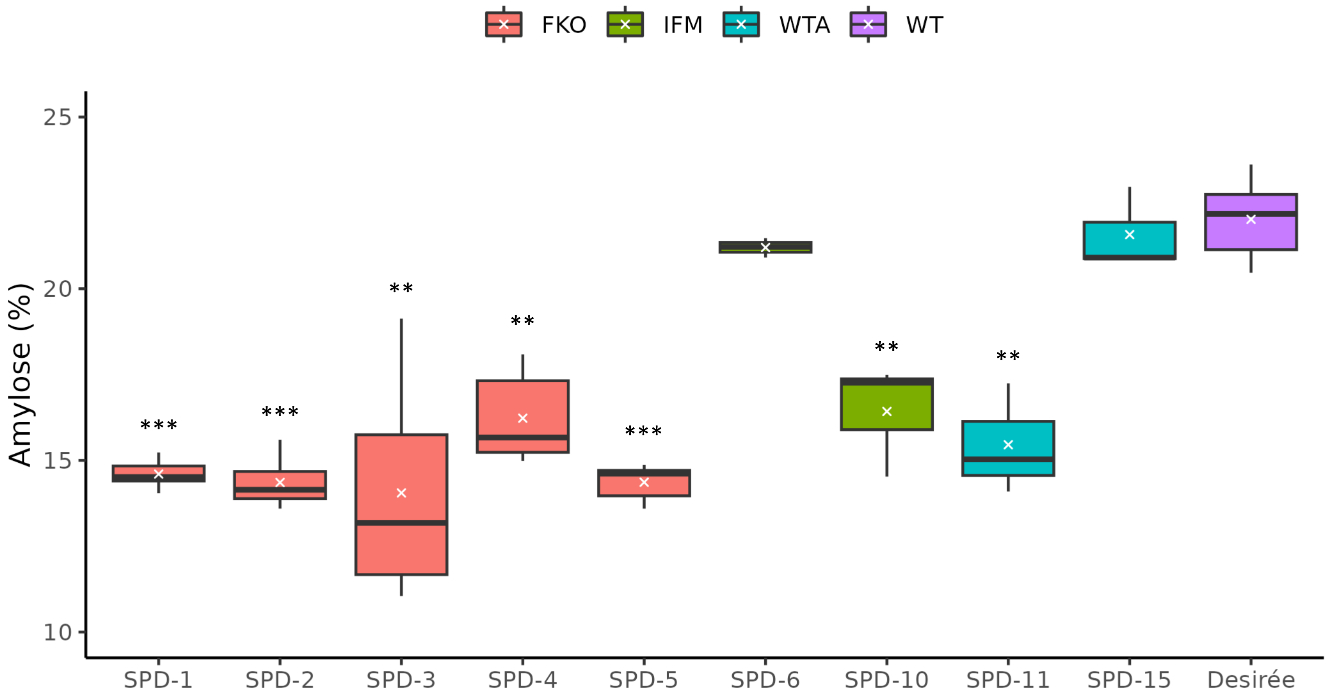

Amylose content in tubers of 9 mutational events and Desirée (WT, control).
Amylose fraction is represented as percentage of dry weight. All measurements were made in triplicates. Average and median values are represented as 'X' and horizontal black bars. Individual events are marked on x axis and colored as per mutational group, i.e., orange: (Full knockouts; FKO), green: (In-Frame knockouts; IFM), blue: (Partial knockouts; WTA) and purple: (control; WT). (* = p<0.05, ** = p<0.01, *** = p<0.001; *t-test* - one tailed, two samples, equal variance)
