# Supplementary material for: Pho1a (plastid starch phosphorylase) is duplicated and essential for normal starch granule phenotype in tubers of Solanum tuberosum L
Source: Front Plant Sci. 2023 Aug 9;14:1220973. doi: 10.3389/fpls.2023.1220973 (PMC10450146; doi:10.3389/fpls.2023.1220973)
Supplement: Supplementary file 7 [file DataSheet_7.pdf]

Supplementary Figure 7:

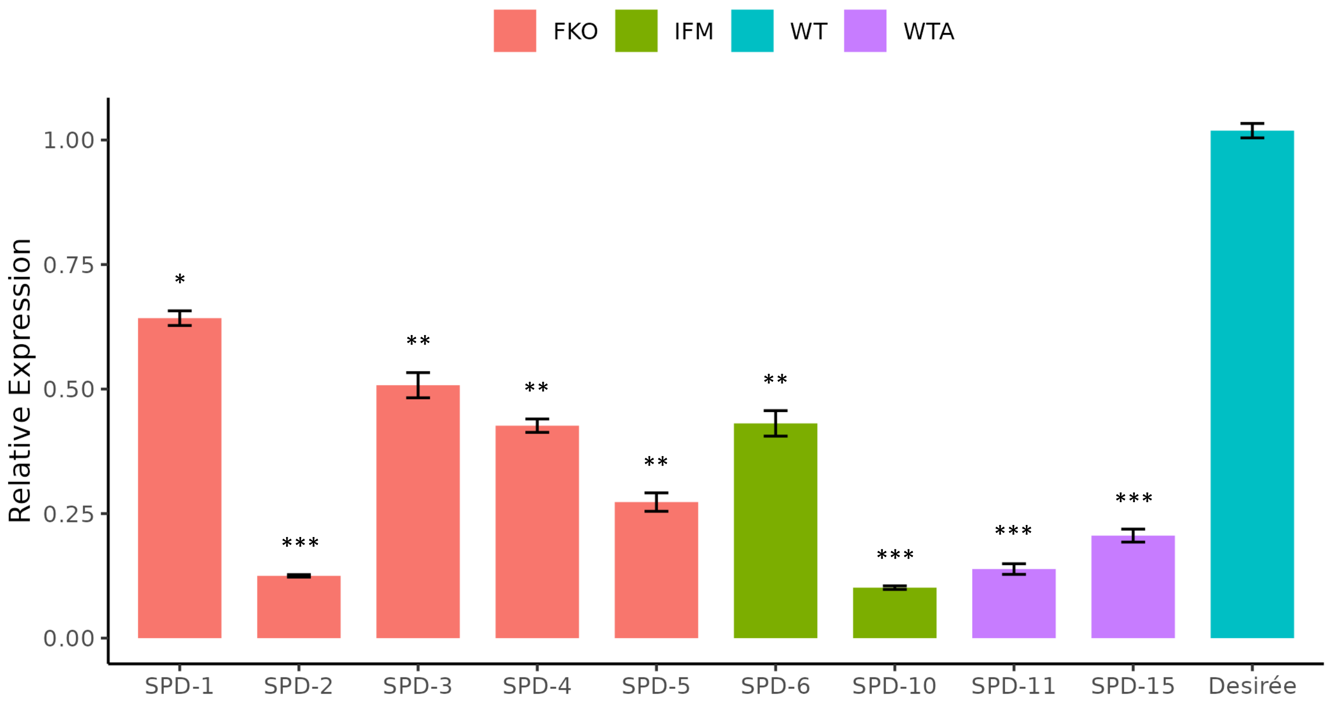

Relative expression of *StGBSS1* in tubers of 9 mutational events and Desirée (WT, control). Relative expression of *StGBSS1* is determined using primers listed in Table 1 and *StTUBB1* (NM_001288449.1) as reference gene. The samples were run in triplicates and the fold change was calculated by $2^{-\Delta\Delta Ct}$. Individual events are marked on x axis and colored as per mutational group, i.e., orange: (Full knockouts; FKO), green: (In-Frame knockouts; IFM), blue: (Partial knockouts; WTA) and purple: (control; WT). (* = p<0.05, ** = p<0.01, *** = p<0.001; *t-test* - one tailed, two samples, equal variance)
