# Supplementary material for: Pho1a (plastid starch phosphorylase) is duplicated and essential for normal starch granule phenotype in tubers of Solanum tuberosum L
Source: Front Plant Sci. 2023 Aug 9;14:1220973. doi: 10.3389/fpls.2023.1220973 (PMC10450146; doi:10.3389/fpls.2023.1220973)
Supplement: Supplementary file 8 [file DataSheet_8.pdf]

Supplementary Figure 8:

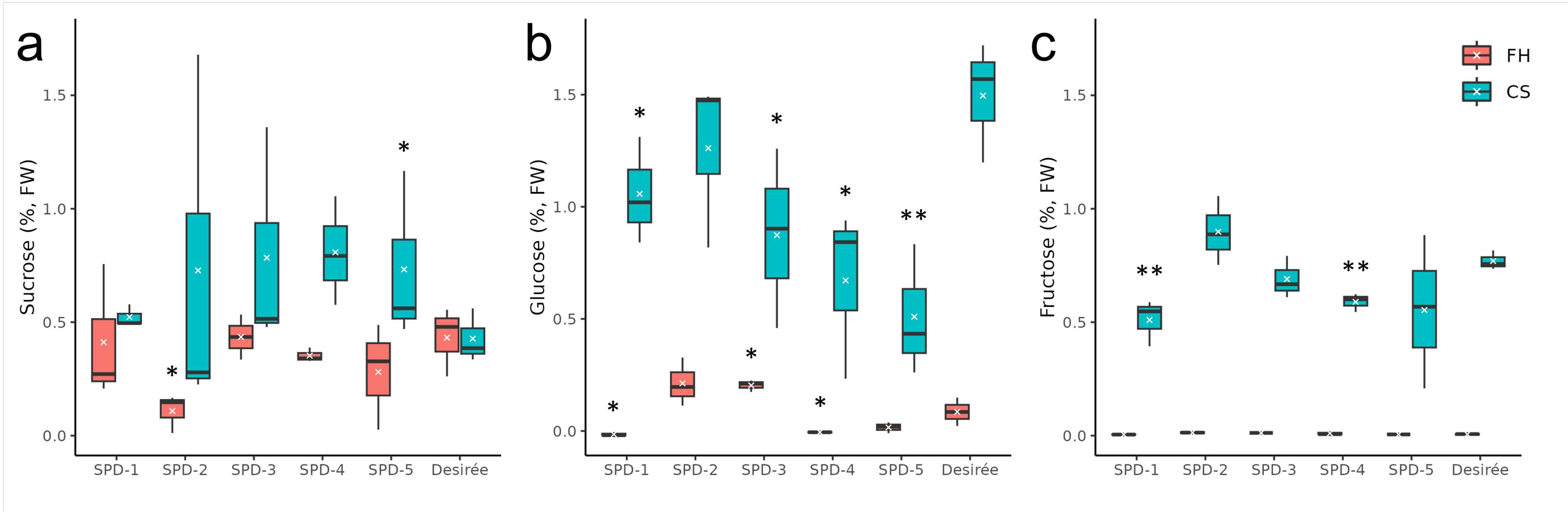

Free sugar levels in fresh and cold-stored tubers of 5 individual full knockout events from Group 1 (FKO) and Desirée (WT, control). a) sucrose, b) glucose and c) fructose levels; represented as percentage of dry weight on respective y axis. Fresh and cold-stored tuber samples are colored as orange and green respectively. All measurements were recorded as triplicates. Statistical analyses were made on freshly harvested tubers and cold stored tubers respectively. Average and median values are represented as 'X' and horizontal black bars. (* = $p<0.05$ and ** = $p<0.01$; t-test - one tailed, two samples, equal variance)
